# Supplementary material for: Study protocol for a non-randomised controlled trial: Community-based occupational therapy intervention on mental health for people with acquired brain injury (COT-MHABI)
Source: PLoS One. 2022 Oct 7;17(10):e0274193. doi: 10.1371/journal.pone.0274193 (PMC9543977; doi:10.1371/journal.pone.0274193)
Supplement: S1 Appendix — (PDF) [file pone.0274193.s002.pdf]

STUDY PROTOCOL FOR A NON-RANDOMISED CONTROLLED TRIAL: A COMMUNITY-BASED OCCUPATIONAL THERAPY INTERVENTION ON MENTAL HEALTH FOR PEOPLE WITH ACQUIRED BRAIN INJURY (COT-MHABI)

Appendix S1

I. DESCRIPTION OF COT-MHABI PROCESS LEVELS, STAGES AND INTERVENTIONS

a) OCCUPATIONAL EXPLORATION LEVEL

General aims:

- Favour the establishment of the therapeutic bond.
- Facilitate participation in meaningful activities after the ABI (previous reconnection/new exploration) in the person's home and community environment.
- Promote access to new opportunities for experimentation and choice of occupational possibilities congruent with volition, performance capacity and the physical and social environment.
- Encourage remaining volition and favor optimisation in environmental exploration.
- Promote access to initial experiences of group participation (social and family) in a safe environment.

Specific aims by stage (summary):

- VALIDATION: Enable access to initial experiences of ability through the use of meaningful activity in a safe environment.
- WILLINGNESS TO EXPLORE: Encourage an optimal basal state to allow for environmental exploration.
- CHOICE: Enable the person to increase his or her sense of capability during exploration and choice of new habits and roles.
- EFFECTIVENESS: Promote continued development of the person's sense of efficacy through exploration and preliminary participation in meaningful habits and roles.

STAGE AIMS: VALIDATION	INTERVENTION CORRESPONDENCE	STAGE AIMS SUMMARY
· Enable access to initial experiences of capacity through the use of meaningful activity.	E2-4	Enable access to initial experiences of capability through the use of meaningful activity in a safe environment.
· Establish a space for mediation and communication between family and person.	E5	
· Facilitate security in the environment (social, family and physical) for the promotion of occupational exploration.	E5-6	
STAGE AIMS: WILLINGNESS TO EXPLORE		
· Promote an optimal baseline state to enable environmental exploration.	E7-11	Favour an optimal basal state to allow environmental exploration.
· Provide support to the social environment to facilitate safe exploration.	E12	
STAGE AIMS: CHOICE		
. Promote an increase in the person's sense of capability through the emergence of self-validation.	E13-15	Enable the person to increase his or her sense of capability during the exploration and selection of new habits and roles.
· Facilitate the exploration and selection of new habits and roles.	E16,17	
· Facilitate support from the social and family environment to the choices made by the person.	E18	
STAGE AIMS: EFFECTIVENESS		
· Facilitate preliminary participation in new habits and chosen roles.	E19,20	Facilitate the individual's continued development of a sense of effectiveness through exploration and preliminary participation in meaningful habits and roles.
· Facilitate conscious reconstruction of occupational identity through self-validation.	E21-22	
· Encourage positive response from the familiy and/or social environment to ensure a sense of efficacy and continuity in exploration.	E23-24	

STAGES	INTERVENTIONS	
VALIDATION	E1<br>E2<br>E3<br>E4<br>E5<br>E6	Initial analysis of volitional components, habituation, objective components (motor, processing, communication and sensory skills) and subjective aspects (lived body).<br>Accompaniment and support in initial experiences of capacity in assumable and meaningful occupational forms.<br>Emotional support in the expression of difficulties and efforts in relation to the lived body and performance skills.<br>Counsel the person in the management of aspects of ABI and MH in relation to occupational participation.<br>Facilitate the expression of demands in the family and/or social interaction spaces.<br>Provide the social and family environment with counselling on aspects and difficulties inherent to ABI and MH.
WILLINGNESS TO EXPLORE THE ENVIRONMENT	E7<br>E8<br>E9<br>E10<br>E11<br>E12	Physical and/or emotional support for the exploration of new occupational actions, spaces, objects and relationships.<br>Counselling and training in the use of support products.<br>Counselling and training in autonomous management of medication intake.<br>Provide strategies to promote autonomy in the management of difficulties related to mental well-being and post-ABI sequelae.<br>Counselling and support to favour autonomy in the management of difficulties related to the consumption of toxic substances.<br>Provide counselling and space for environment-person dialogue.
CHOICE	E13<br>E14<br>E15<br>E16<br>E17<br>E18	Provide strategies for self-analysis and self-validation of present volitional aspects.<br>Accompaniment in decision-making in relation to significant volitional aspects.<br>Facilitate exploration of new volitional opportunities according to social and community environment.<br>Facilitate strategies for the establishment of new routines.<br>Facilitate increased time spent and frequency of meaningful routines.<br>Provide the family environment with strategies to facilitate its adaptation to the person's significant choices.
PLEASURE AND EFFECTIVENESS IN ACTION	E19<br>E20<br>E21<br>E22<br>E23<br>E24	Facilitate accessibility to occupational forms and tasks of the chosen routines and roles.<br>Provide strategies to increase participation in daily and weekly routines.<br>Facilitate the emergence of the process of inscription of the sense of efficacy through positive feedback.<br>Facilitate through feedback the self-analysis of performance capabilities.<br>Provide feedback to the family and/or social environment on progress achieved by the person in the different stages of exploration.<br>Provide strategies to the family and/or social environment to increase effectiveness in the person's performance.

b) OCCUPATIONAL COMPETENCE LEVEL

General aims:

- Enable the person to plan and execute challenges (congruent with performance and the environment) in his or her occupational participation to increase the sense of effectiveness, with the support of the family and/or social environment.
- Promote an increase in the person's capacity expectations in performance to facilitate skills development.
- Accompany the person in the process of building his or her occupational narrative, reinforcing processes of building a new desired occupational identity.

Specific aims by stage (summary):

- INTERNALISING A SENSE OF EFFECTIVENESS: Promote self-analysis of capacity and effectiveness in performance, planning challenges and objectives, in congruence with aspects of habituation (habits and roles) and the family and/or social environment. Promote the acquisition of management strategies to face the difficulties present in his or her occupational performance.
- OCCUPATIONAL NARRATIVE BUILDING: Facilitate the performance of occupational actions and roles that develop and improve skills (motor, processing and communication and interaction) affected by ABI. Facilitate the family and/or social environment's autonomy in the care and accompaniment of the evolution of occupational competence. Facilitate registration of the new occupational narrative of continuity (role project).

STAGE AIMS: INTERNALISING A SENSE OF EFFECTIVENESS	INTERVENTION CORRESPONDENCE	STAGE AIMS SUMMARY
· Facilitate access to challenging occupational opportunities according to congruence with volitional aspects, roles and performance capacity.	C1	Foster self-analysis of capacity and effectiveness in performance, planning challenges and objectives, in congruence with aspects of habituation (habits and roles) and the family and/or social environment. Promote the acquisition of management strategies to face the difficulties present in his or her occupational performance.
· Help the person to carry out the approach of new goals and objectives in an autonomous way.	C2	
· Favour the establishment of routines and habits in relation to desired roles and performance capacity.	C3,4	
· Facilitate the emergence of management strategies in performance.	C5,6	
· Facilitate support from the family and social environment to favour an increased sense of effectiveness and support role performance.	C7-9	
STAGE AIMS: OCCUPATIONAL NARRATIVE BUILDING		
· Facilitate autonomous continuity of meaningful occupational actions.	C10-13	Facilitate the performance of occupational actions and roles that develop and improve skills (motor, processing and communication and interaction) affected by ABI. Facilitate the family and/or social environment's autonomy in the care and accompaniment of the evolution of occupational competence. Facilitate registration of the new occupational narrative of continuity (role project).
· Facilitate the performance of occupational actions and roles that develop and improve skills (motor, processing, communication and interaction) affected by ABI and necessary for occupational participation.	C14-15	
· Provide support in the expression of the lived body and its place in the occupational continuum.	C17	
· Facilitate the inscription of a new narrative of continuity (role project) in relation to occupational performance.	C17	
· Increase family members' capacity to manage overload.	C18,19	
· Provide support in the expression of the impact of ABI and its inscription in the occupational continuum within the life history of the family system.	C19	

STAGES	INTERVENTIONS	
INTERN. SENSE OF EFFECTIVENESS	C1	Support and accompaniment in the gradation and experimentation of activities of daily living (ADLs) and leisure to facilitate occupational challenge.
	C2	Accompaniment in the autonomous updating of the objectives of the process.
	C3	Facilitate spatial-temporal structuring of daily tasks and occupations.
	C4	Facilitate self-analysis of effectiveness in the performance of routines and habits.
	C5	Facilitate optimisation of the use of objective and subjective strengths in occupational performance.
	C6	Counselling on management strategies in the face of performance difficulties.
	C7	Facilitate the family and/or social environment's understanding of the person's strengths and weaknesses in relation to performance.
	C8	Facilitating the family and/or social environment's ability to provide positive feedback to the person on his or her occupational performance.
	C9	Provide advice to the family and/or social environment to optimise the ability to detect and assess effectiveness milestones and their importance in the rehabilitation process.
OCCUPATIONAL NARRATIVE BUILDING.	C10	Facilitate self-analysis of volitional milestones.
	C11	Facilitate the continuity of the process of inscribing a sense of effectiveness through positive feedback.
	C12	Facilitate self-analysis of effective/non-effective performance.
	C13	Facilitate resolution and management strategies in the face of unsuccessful occupational performance actions.
	C14	Provide advice and support for the realisation of occupational actions that develop and improve motor skills affected by ABI.
	C15	Provide advice and support for the realization of occupational actions that develop and improve processing skills affected by ABI.
	C16	Provide advice and support for the implementation of occupational actions that develop and improve communication and interaction skills affected by ABI.
	C17	Provide space and/or tools for listening, written expression or creative mediation for self-recognition of the process.
	C18	Provide the social/family environment with tools for self-care and self-analysis of occupational balance.
	C19	Provide space and/or tools for listening, written expression or creative mediation for caregiver overload management.

c) OCCUPATIONAL ACHIEVEMENT LEVEL

General aims:

- Facilitate autonomy in setting personal goals and meaningful occupational choices.
- Facilitate continuity of learning of critical skills for desired occupational participation and the establishment of new occupational challenges.

Specific aims by stage (summary):

- Facilitate internalisation of habits and execution of significant roles and consolidation of occupational actions that improve occupational performance.
- Optimise the occupational balance of the person and family environment in relation to the new occupations achieved.
- Facilitate information and support for the continuity of performance in the post-intervention phase.

STAGE AIMS	INTERV. CORRESP.	STAGE AIMS SUMMARY
· Facilitate internalisation of habits and execution of roles congruent with the volitional aspects of the person.	L1	· Facilitate internalisation of habits and execution of significant roles and consolidation of occupational actions that improve occupational performance.<br>· Optimise the occupational balance of the person and family environment in relation to the new occupations achieved.<br>· Facilitate information and support for the continuity of performance in the post-intervention phase.
· Facilitate information and support for the continuity of performance in the post-intervention phase.	L4	
· Facilitate consolidation of occupational actions that develop and increase performance capabilities.	L5-9	
· Provide support to the family and/or social environment to favour autonomy and independence in the performance of meaningful occupations and roles.	L10-13	
· Optimise the occupational balance of the family environment in relation to the new occupations achieved by the person.	L11	

INTERVENTIONS	
L1	Provide positive feedback for the internalisation of habits.
L2	Provide preventive strategies for obstacles to achievement in occupational participation.
L3	Provide strategies for the minimisation and/or management of unavoidable obstacles.
L4	Provide information and support for continued performance in the post-intervention phase.
L5	Provide advice and support in occupational actions to improve motor skills affected by ABI.
L6	Provide advice and support in occupational actions that improve processing skills affected by ABI.
L7	Provide advice and support in occupational actions to improve communication and interaction skills affected by ABI.
L8	Facilitate space and/or tools for listening, written expression or creative mediation for the inscription of a diverse functional narrative of the experienced body.
L9	Support in the emotional management of the occupational impact of ABI and the symptomatology of mental disorder.
L10	Enable the family and/or social environment to recognise occupational milestones and provide subsequent positive feedback to the person.
L11	Facilitate self-analysis strategies of the occupational balance of the social environment and family system.
L12	Provide the social and/or family environment with strategies for the continuity of achievements.
L13	Provide the social and/or family environment with prevention skills in self-care and future care of the family member.
