# Supplementary material for: Study protocol for a non-randomised controlled trial: Community-based occupational therapy intervention on mental health for people with acquired brain injury (COT-MHABI)
Source: PLoS One. 2022 Oct 7;17(10):e0274193. doi: 10.1371/journal.pone.0274193 (PMC9543977; doi:10.1371/journal.pone.0274193)
Supplement: S1 File — (PDF) [file pone.0274193.s003.pdf]

# PROPOSAL

## Community-based Occupational Therapy Intervention on Mental Health for People With Acquired Brain Injury (COT-MHABI)

# INTRODUCTION

Acquired brain injury (ABI) is defined as brain injury after birth, resulting from an external force (as in the case of traumatic brain injury) or due to non-traumatic processes (stroke, anoxia, brain tumors, encephalitis, etc.) (1). The sequelae of moderate-severe ABI encompass motor, cognitive, sensory, emotional and behavioural areas that affect occupational participation, social relationships and quality of life, often resulting in personality changes (2–7).

This population profile shows a significantly high prevalence of associated mental disorders, either due to exacerbation of pre-existing symptoms, due to the onset of organic injury, or due to the resulting psychosocial situation (8–10). However, on returning to the community after the hospital stage, the specialised care they receive is generally insufficient, being treated from a psychiatric rather than neurological perspective, or directly rejected by these services (11–14), mainly due to lack of knowledge of the relationship between ABI and mental health and the invisibility of many of the associated deficits (15–17). Thus, people with moderate-severe ABI have, according to the World Health Organization (WHO), a highly complex condition, since it manifests deficits in bodily functions, activity limitation and restriction in participation in life situations (18).

One of the most appropriate disciplines to support survivors in managing the consequences of the deficits produced by ABI is occupational therapy (19). The Model of Human Occupation (MOHO), specific to occupational therapy, is the most widely used occupation-focused model in the world (19). It is a person-centred, evidence-based model that provides specific assessments and interventions focused on meaningful occupation. It is based on four main aspects: motivation for occupation, habits and routines, occupational performance skills, and the influence of the environment on participation (19–21). The structure provided by the MOHO facilitates access to an intervention focused on the recovery of meaningful occupations for the person, accompanying the exploration of new participation options, the performance of roles and the active reconstruction of an identity narrative interfered with by the deficits and their behavioural, emotional and social correlates (7,19,20,22,23).

# MATERIALS AND METHODS

## Objectives

The aim of this study is to: (A) design a protocol to evaluate the effectiveness of a community occupational therapy intervention based on MOHO in mental health for people with ABI, developed in a home setting and focused on meaningful occupation; (B) know the contribution to quality of life of satisfaction with occupations performed by a group of people with ABI and mental disorder.

## Inclusion and exclusion criteria

The study population (experimental and control group) will be composed of adults over 18 years of age with a diagnosis of medium or severe ABI and a diagnosis of neurocognitive disorder following ABI (as recognised in the 5th edition of the Diagnostic and Statistical Manual of Mental Disorders (DSM-5); by definition: evidence of significant cognitive decline from pre-ABI level in one or more cognitive domains (attention, executive function, learning and memory, perceptual-motor ability or social cognition). It may occur with or without behavioural impairment (apathy, mood disturbance, irritability, disinhibition, psychotic symptoms, etc.)). This population (A) show difficulties in occupational participation with respect to the pre-ABI situation and present needs for support, counselling and/or specific therapeutic intervention; (B) are in a situation of hospital discharge from ABI specialisation units; (C) are domiciled in the same province to which the providing hospital belongs. In addition, they may or may not have been diagnosed with a mental health disorder other than neurocognitive disorder, either prior or subsequent to the ABI.

Persons will be excluded if (A) they are in a situation of symptomatological destabilisation of severe functional impairment that, as a priority, requires continued support from specialised mental health units, psychiatric or social-health care admission; and/or (B) they are unable to determine for themselves (for cognitive reasons or by conscious choice) any problem in at least one occupational area.

## Recruitment methods

The intervention provided for the experimental group is framed within the community intervention programme of Occupational Therapy in mental health and ABI developed at Institut Guttmann Neurorehabilitation Hospital in Badalona (Spain). As it is a very specific and novel intervention, the resources for its implementation are limited, generating a waiting list. Given that this standby period is longer than the duration of the intervention in the experimental group, patients on the waiting list will be invited to participate in the control group, with the consideration of receiving the intervention at a later stage.

For both the experimental and control groups, the referral of patients to the study is done through the neuropsychiatry department of the hospital, which does not participate in the intervention, data collection or statistical analysis of the results. In addition, the professionals responsible for the data collection will not be involved in the allocation of subjects.

## Trial design and setting

A non-randomised controlled clinical trial will be performed, due to the logistical reasons discussed above. The measurement points of both groups will be comparable, matching gender, age, geographical area of residence and diagnosis. Fig.1 includes a flow chart of the main parts of the study.

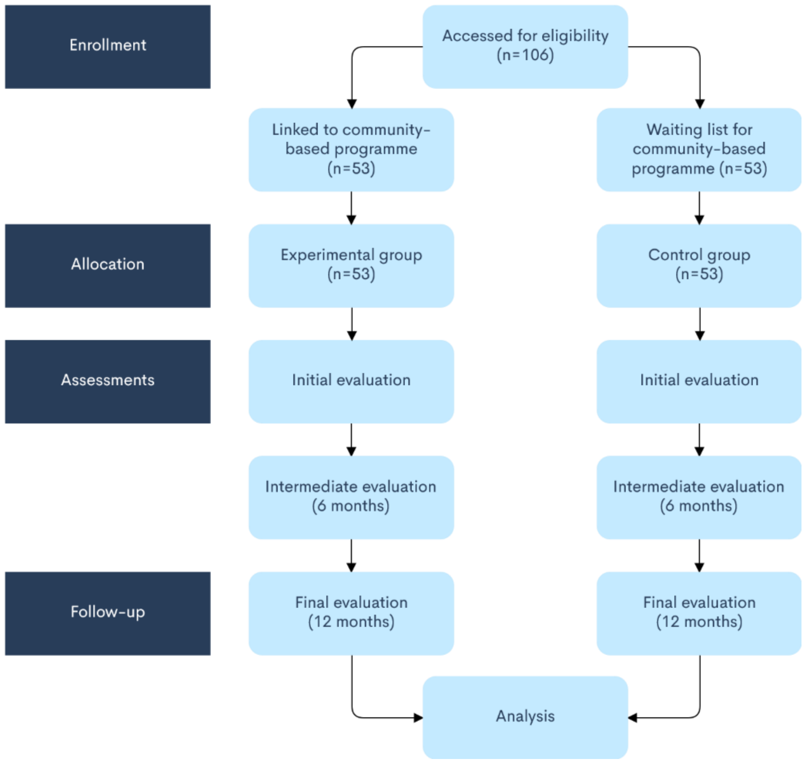


*Fig. 1*

## Intervention

### Experimental group: community-based occupational therapy intervention in mental health for people with ABI

Participants in the experimental arm will receive a community occupational therapy intervention in mental health, which will take into account the ABI's related issues, carried out in a home setting and with meaningful occupation as the main focus. The intervention process, in general, will follow the diagram shown in Fig. 2.

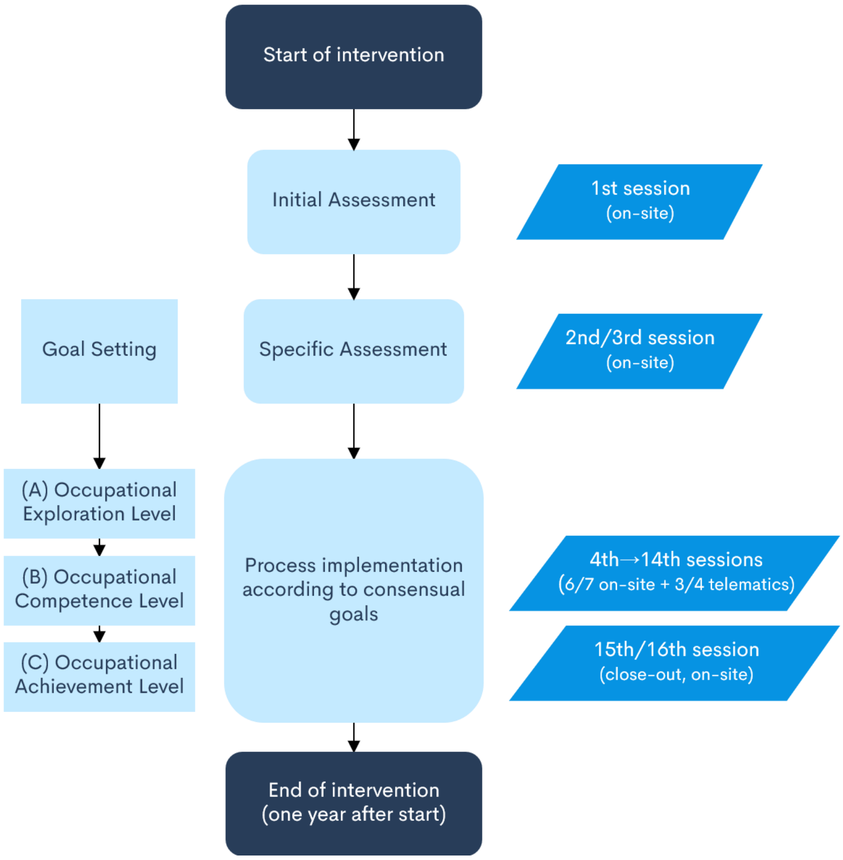


*Fig. 2*

Once the person accepts to participate in the study, an information sheet will be handed out and the consent forms will be signed. The principal investigator will carry out the intervention and the different evaluations of the study.

The occupational therapy sessions will be implemented through an occupation-based and occupation-focused intervention (24) whose structure comes from an adaptation of the Model of Human Occupation (MOHO) Remotivation Process (21,25), which has as one of its main objectives the facilitation of participation in occupations in people with severe volitional difficulties (21).

During the one-year process, a maximum of 12 on-site sessions and 4 synchronous telematic sessions in video-call format are foreseen. The on-site sessions, which will take place in a home and/or community environment, will last between 60 and 90 minutes, depending on the objectives set, and the telematic sessions will last approximately 45 minutes.

In the exceptional case in which it is not possible to carry out the session in person or by video-call, it will be carried out by telephone call. Likewise, both telephonic and asynchronous communication (e-mail or text messages) will be considered for possible exchanges of information or contingencies, without these communications constituting an intervention session per se.

*Development of the sessions*

During the first session, the initial comprehensive assessment will be carried out, where the occupational therapist will collect information regarding the occupational history, i.e. significant activities for the person and their impact on the life narrative, and about current occupational participation, i.e. the occupations that the person is effectively performing at the time of the assessment.

During the second session, a specific assessment will be carried out, where a detailed analysis of relevant areas and facilitators/hinderers of occupational participation will be performed, together with an exploration of significant roles and the person's expectations about them. Based on this information and the information gathered in the first session, the objectives will be established with the person's agreement.

In order to achieve the objectives, occupational therapy interventions will be carried out during the rest of the process, which will be defined on the basis of three sequential and interrelated levels that correspond to the continuum of change defined by the MOHO (21,25). Following the structure of the previously mentioned Remotivation Process of the model, the following levels are considered: (A) Occupational Exploration; (B) Occupational Competence; and (C) Occupational Achievement. Each level corresponds to a series of stages composed, in turn, of intervention strategies. A schematic summary of the composition of these levels can be seen in Table 1, while the complete list of strategies and goals corresponding to each level appears in the Appendix.

*Table 1. Composition of the intervention process levels*

| Levels | Stages | Stage goals | Intervention strategy example |
|---|---|---|---|
| Occupational exploration | Validation | Enable access to initial experiences of capability through meaningful activity in a safe environment. | Accompaniment and support in initial experiences of capacity in acceptable and meaningful occupational forms. |
| | Willingness to explore | Favour an optimal basal state for allowing environmental exploration. | Provide strategies for promoting autonomy in managing difficulties related to mental well-being and post-ABI deficits. |
| | Election | Enable the person to increase his or her sense of capability during the exploration and choice of new habits and roles. | Facilitate exploration of new volitional opportunities according to the social and community environment. |
| | Effectiveness | Promote continued development of the person's sense of efficacy through exploration and preliminary participation in meaningful habits and roles. | Facilitate accessibility to occupational forms and tasks appropriate to the routines and chosen roles. |
| Occupational Competence | Internalisation of a sense of effectiveness | Foster self-analysis of capacity and effectiveness in performance, planning challenges and objectives, in congruence with aspects of habituation (habits and roles) and the physical and social environment.<br>Promote the acquisition of management strategies to face the difficulties present in his/her occupational performance. | Provide counselling to the family and social environment to optimise the ability to detect and assess effectiveness milestones and their importance within the rehabilitation process. |
| | Occupational narrative building | Facilitate the realisation of occupational actions and roles that develop and improve affected skills (motor, processing, communication and interaction). Facilitate registration of the new occupational narrative of continuity (role project). | Facilitating the continuity of the process of inscribing a sense of effectiveness through positive feedback. |
| OccupationalA chievement | Achievement | Facilitate internalisation of habits and execution of meaningful roles and consolidation of occupational actions that improve occupational performance. | Provide preventive strategies for barriers to achievement in occupational participation. |

| | | |
|---|---|---|
| | | Optimise the occupational balance of the person and family environment in relation to the new occupations achieved.<br>Provide information and support for continued performance in the post-intervention phase. |

Thus, the general proposal of the intervention process will be to carry out a progressive path that enables the person to experience how to: (A) explore new occupations through the development of awareness of one's own capacity in a safe environment; (B) integrate these new learnings into habits congruent with the demands of the roles and the physical and social environment, experiencing an increase in personal effectiveness; (C) carry out an effective increase in occupational participation that has a permanent impact on occupational identity, in congruence with the pattern of life desired by the person. These interventions will be aimed at seeking personal autonomy through the use of meaningful activity, the adaptation of the physical environment and the optimisation of the relationship with the social and family environment (21,25).

The therapeutic intervention process will be complemented with counselling actions in relation to ABI and mental health for (social and health) care resources and, in general, for community agents involved in the planning of occupational participation of the person. Likewise, actions will be carried out to connect resources required by the intervention, always with the aim of facilitating an optimisation of occupational performance that fosters participation and occupational balance, both for the person and the family environment.

### Control group: regular health care treatments (private/public health services)

The people belonging to the control group will receive the usual services of their health centers, mainly access to outpatient consultations and, in some cases, private treatments. The duration of these treatments will be estimated by the centers themselves, depending on the patient's clinical condition and/or demand.

## Data collection

The variables to be collected in the study will be evaluated at three points in time: initial evaluation, intermediate evaluation at six months of the process, and final evaluation at twelve months. The scales used and their temporality will be the same for both the experimental group and the control group. The collection will be carried out by different researchers.

## Main outcomes

### Quality of life

This will be measured by the WHOQoL-BREF (World Health Organization Quality of Life-BREF, Spanish version) (26,27). This is a generic self-administered questionnaire created by the Quality of Life Study Group of the World Health Organization (WHO). The instrument has 26 questions, two general questions on quality of life and satisfaction with health status, and 24 questions grouped into four areas: Physical Health, Psychological Health, Social Relationships and Environment. Higher scores indicate better perceived quality of life. A 5-point Likert-type response scale is used.

### Perceived occupational performance and satisfaction with performance

This will be evaluated by COPM (Canadian Occupational Performance Measure, Spanish version) (21,28), which is a self-administered occupational therapy measure based on client-centred practice that helps to establish occupational needs. It assesses changes in self-perceived occupational performance and satisfaction with occupational performance following intervention.

## Secondary outcomes

### Satisfaction with occupations and occupational balance

This will be measured using the SDO-OB scale (Satisfaction with Daily Occupation and Occupational Balance, SDO-OB, Spanish version (SOD-EO)) (29,30). The instrument assesses satisfaction in thirteen occupational areas, organised in four domains (productivity, leisure, housework and self-care) in terms of the person's activity level, occupational satisfaction and balance. Satisfaction level scores are answered based on a 7-item scale.

### Participation and satisfaction with the performed roles

This will be evaluated by RCv3 (Role Checklist Version 3: Participation and Satisfaction) (31). The instrument assesses the person's perception of role performance, role satisfaction, and desire to participate in other roles in the future.

### Self-perceived level of participation in activities

This will be measured using the ACS scale (Activity Card Sort, Spanish version) (32). This is an instrument composed of a total of 89 photographs, used in occupational therapy to perform a joint exploration with the person, noting a catalogue of activities performed in all areas, both before and after the injury or situation that interferes with performance. It is useful to identify loss of participation, set goals and analyse evolution. The items include 20 instrumental activities, 17 social activities and 35 leisure activities of low physical demand and 17 of high demand.

### Community integration

This will be measured from the results collected in the CIQ (Community Integration Questionnaire) (33). It assesses the limitations in the performance of social roles and community interaction of people with ABI. It presents three main dimensions: domestic integration, social integration and productivity. The total score range is between 0-29 and most items have a range of 0-2. Higher values represent greater community integration and independence.

### Functional independence

This will be valued from the FIM scale (Functional Independence Measure) (34,35). The scale represents a uniform measurement system for disability based on the International Classification of Impairment, Disabilities and Handicaps. It assesses six functional areas (self-care, sphincter control, transfers, locomotion, communication and social cognition) within two domains (motor and cognitive). Each item is scored on a 7-point Likert-type scale. The items are performance-based rather than ability-based and are recorded in a hetero-administered manner.

### Satisfaction with the intervention process

This will be collected qualitatively and quantitatively by regular programme satisfaction questionnaires. In this case, there is one questionnaire for the person participating in the process and another one for the main referring family member, if any. Both questionnaires are composed of seven questions about the subjective perception of the results obtained after the intervention with a 7-point Likert-type response scale. In addition, there are 4 more questions that, with a scale of 1 to 10, aim to collect the opinion of the quality of the intervention. Finally, a section will be included where, in a qualitative manner, the person can express other comments not included in the previous questions.

## SCHEDULE

Table 3. Schedule

| | | | DATES | | | | |
|---|---|---|---|---|---|---|---|
| Tasks | Institutional reference | Investigators | 2019 | 2020 | 2021 | 2022 | 2023 |
| DESIGN | | | | | | | |
| Idea conception and study design | UVic-IG | MARR - BCM-JAMB | X | | | | |
| Planning | UVic-IG | MARR - BCM-JAMB | X | X | X | | |
| Design redefinition | UVic-IG | MARR - BCM-JAMB | | X | X | | |
| INTERVENTION: COLLECTION AND FOLLOW-UP | | | | | | | |
| Clinical data collection | UVic-IG | MARR - BCM-JAMB | | X | X | X | |
| ANALYSIS | | | | | | | |
| Data Processing | UVic-IG | MARR - BCM-JAMB | | | | X | |
| Statistic analysis | UVic-IG | MARR - BCM-JAMB | | | | X | |
| DISSEMINATION | | | | | | | |
| Conferences, meetings | IG | MARR - BCM-JAMB | | | X | X | X |
| Scientific papers | UVic-IG | MARR - BCM-JAMB | | | X | X | X |

UVic: Universitat de Vic – Universitat Central de Catalunya; IG: Institut Guttmann

MARR: Marco Antonio Raya-Ruiz; BCM: Beatriz Castaño-Monsalve; JAMB: Jose Antonio Merchán-Baeza

# APPENDIX

## I. DESCRIPTION OF PROCESS LEVELS, STAGES AND INTERVENTIONS

### a) OCCUPATIONAL EXPLORATION LEVEL

General aims:

- Favour the establishment of the therapeutic bond.
- Facilitate participation in meaningful activities after the ABI (previous reconnection/new exploration) in the person's home and community environment.
- Promote access to new opportunities for experimentation and choice of occupational possibilities congruent with volition, performance capacity and the physical and social environment.
- Encourage remaining volition and favor optimisation in environmental exploration.
- Promote access to initial experiences of group participation (social and family) in a safe environment.

Specific aims by stage (summary):

- VALIDATION: Enable access to initial experiences of ability through the use of meaningful activity in a safe environment.
- WILLINGNESS TO EXPLORE: Encourage an optimal basal state to allow for environmental exploration.
- CHOICE: Enable the person to increase his or her sense of capability during exploration and choice of new habits and roles.
- EFFECTIVENESS: Promote continued development of the person's sense of efficacy through exploration and preliminary participation in meaningful habits and roles.

| STAGE AIMS: VALIDATION | INTERVENTION CORRESPONDENCE | STAGE AIMS SUMMARY |
|---|---|---|
| · Enable access to initial experiences of capacity through the use of meaningful activity. | E2-4 | Enable access to initial experiences of capability through the use of meaningful activity in a safe environment. |
| · Establish a space for mediation and communication between family and person. | E5 | |
| · Facilitate security in the environment (social, family and physical) for the promotion of occupational exploration. | E5-6 | |
| STAGE AIMS: WILLINGNESS TO EXPLORE | | |
| · Promote an optimal baseline state to enable environmental exploration. | E7-11 | Favour an optimal basal state to allow environmental exploration. |
| · Provide support to the social environment to facilitate safe exploration. | E12 | |
| STAGE AIMS: CHOICE | | |
| . Promote an increase in the person's sense of capability through the emergence of self-validation. | E13-15 | Enable the person to increase his or her sense of capability during the exploration and selection of new habits and roles. |
| · Facilitate the exploration and selection of new habits and roles. | E16,17 | |
| · Facilitate support from the social and family environment to the choices made by the person. | E18 | |
| STAGE AIMS: EFFECTIVENESS | | |
| · Facilitate preliminary participation in new habits and chosen roles. | E19,20 | Facilitate the individual's continued development of a sense of effectiveness through exploration and preliminary participation in meaningful habits and roles. |
| · Facilitate conscious reconstruction of occupational identity through self-validation. | E21-22 | |
| · Encourage positive response from the familiy and/or social environment to ensure a sense of efficacy and continuity in exploration. | E23-24 | |

| STAGES | INTERVENTIONS | |
|---|---|---|
| VALIDATION | E1 | Initial analysis of volitional components, habituation, objective components (motor, processing, communication and sensory skills) and subjective aspects (lived body). |
| | E2 | Accompaniment and support in initial experiences of capacity in assumable and meaningful occupational forms. |
| | E3 | Emotional support in the expression of difficulties and efforts in relation to the lived body and performance skills. |
| | E4 | Counsel the person in the management of aspects of ABI and MH in relation to occupational participation. |
| | E5 | Facilitate the expression of demands in the family and/or social interaction spaces. |
| | E6 | Provide the social and family environment with counselling on aspects and difficulties inherent to ABI and MH. |
| WILLINGNESS TO EXPLORE THE ENVIRONMENT | E7 | Physical and/or emotional support for the exploration of new occupational actions, spaces, objects and relationships. |
| | E8 | Counselling and training in the use of support products. |
| | E9 | Counselling and training in autonomous management of medication intake. |
| | E10 | Provide strategies to promote autonomy in the management of difficulties related to mental well-being and post-ABI sequelae. |
| | E11 | Counselling and support to favour autonomy in the management of difficulties related to the consumption of toxic substances. |
| | E12 | Provide counselling and space for environment-person dialogue. |
| CHOICE | E13 | Provide strategies for self-analysis and self-validation of present volitional aspects. |
| | E14 | Accompaniment in decision-making in relation to significant volitional aspects. |
| | E15 | Facilitate exploration of new volitional opportunities according to social and community environment. |
| | E16 | Facilitate strategies for the establishment of new routines. |
| | E17 | Facilitate increased time spent and frequency of meaningful routines. |
| | E18 | Provide the family environment with strategies to facilitate its adaptation to the person's significant choices. |
| PLEASURE AND EFFECTIVENESS IN ACTION | E19 | Facilitate accessibility to occupational forms and tasks of the chosen routines and roles. |
| | E20 | Provide strategies to increase participation in daily and weekly routines. |
| | E21 | Facilitate the emergence of the process of inscription of the sense of efficacy through positive feedback. |
| | E22 | Facilitate through feedback the self-analysis of performance capabilities. |
| | E23 | Provide feedback to the family and/or social environment on progress achieved by the person in the different stages of exploration. |
| | E24 | Provide strategies to the family and/or social environment to increase effectiveness in the person's performance. |

b) OCCUPATIONAL COMPETENCE LEVEL

General aims:

- Enable the person to plan and execute challenges (congruent with performance and the environment) in his or her occupational participation to increase the sense of effectiveness, with the support of the family and/or social environment.
- Promote an increase in the person's capacity expectations in performance to facilitate skills development.
- Accompany the person in the process of building his or her occupational narrative, reinforcing processes of building a new desired occupational identity.

Specific aims by stage (summary):

- INTERNALISING A SENSE OF EFFECTIVENESS: Promote self-analysis of capacity and effectiveness in performance, planning challenges and objectives, in congruence with aspects of habituation (habits and roles) and the family and/or social environment. Promote the acquisition of management strategies to face the difficulties present in his or her occupational performance.
- OCCUPATIONAL NARRATIVE BUILDING: Facilitate the performance of occupational actions and roles that develop and improve skills (motor, processing and communication and interaction) affected by ABI. Facilitate the family and/or social environment's autonomy in the care and accompaniment of the evolution of occupational competence. Facilitate registration of the new occupational narrative of continuity (role project).

| STAGE AIMS: INTERNALISING A SENSE OF EFFECTIVENESS | INTERVENTION CORRESPONDENCE | STAGE AIMS SUMMARY |
|---|---|---|
| · Facilitate access to challenging occupational opportunities according to congruence with volitional aspects, roles and performance capacity. | C1 | Foster self-analysis of capacity and effectiveness in performance, planning challenges and objectives, in congruence with aspects of habituation (habits and roles) and the family and/or social environment. Promote the acquisition of management strategies to face the difficulties present in his or her occupational performance. |
| · Help the person to carry out the approach of new goals and objectives in an autonomous way. | C2 | |
| · Favour the establishment of routines and habits in relation to desired roles and performance capacity. | C3,4 | |
| · Facilitate the emergence of management strategies in performance. | C5,6 | |
| · Facilitate support from the family and social environment to favour an increased sense of effectiveness and support role performance. | C7-9 | |
| STAGE AIMS: OCCUPATIONAL NARRATIVE BUILDING | | |
| · Facilitate autonomous continuity of meaningful occupational actions. | C10-13 | Facilitate the performance of occupational actions and roles that develop and improve skills (motor, processing and communication and interaction) affected by ABI. Facilitate the family and/or social environment’s autonomy in the care and accompaniment of the evolution of occupational competence. Facilitate registration of the new occupational narrative of continuity (role project). |
| · Facilitate the performance of occupational actions and roles that develop and improve skills (motor, processing, communication and interaction) affected by ABI and necessary for occupational participation. | C14-15 | |
| · Provide support in the expression of the lived body and its place in the occupational continuum. | C17 | |
| · Facilitate the inscription of a new narrative of continuity (role project) in relation to occupational performance. | C17 | |
| · Increase family members' capacity to manage overload. | C18,19 | |
| · Provide support in the expression of the impact of ABI and its inscription in the occupational continuum within the life history of the family system. | C19 | |

| STAGES | INTERVENTIONS | |
|---|---|---|
| INTERN. SENSE OF EFFECTIVENESS | C1 | Support and accompaniment in the gradation and experimentation of activities of daily living (ADLs) and leisure to facilitate occupational challenge. |
| | C2 | Accompaniment in the autonomous updating of the objectives of the process. |
| | C3 | Facilitate spatial-temporal structuring of daily tasks and occupations. |
| | C4 | Facilitate self-analysis of effectiveness in the performance of routines and habits. |
| | C5 | Facilitate optimisation of the use of objective and subjective strengths in occupational performance. |
| | C6 | Counselling on management strategies in the face of performance difficulties. |
| | C7 | Facilitate the family and/or social environment's understanding of the person's strengths and weaknesses in relation to performance. |
| | C8 | Facilitating the family and/or social environment's ability to provide positive feedback to the person on his or her occupational performance. |
| | C9 | Provide advice to the family and/or social environment to optimise the ability to detect and assess effectiveness milestones and their importance in the rehabilitation process. |
| OCCUPATIONAL NARRATIVE BUILDING. | C10 | Facilitate self-analysis of volitional milestones. |
| | C11 | Facilitate the continuity of the process of inscribing a sense of effectiveness through positive feedback. |
| | C12 | Facilitate self-analysis of effective/non-effective performance. |
| | C13 | Facilitate resolution and management strategies in the face of unsuccessful occupational performance actions. |
| | C14 | Provide advice and support for the realisation of occupational actions that develop and improve motor skills affected by ABI. |
| | C15 | Provide advice and support for the realization of occupational actions that develop and improve processing skills affected by ABI. |
| | C16 | Provide advice and support for the implementation of occupational actions that develop and improve communication and interaction skills affected by ABI. |
| | C17 | Provide space and/or tools for listening, written expression or creative mediation for self-recognition of the process. |
| | C18 | Provide the social/family environment with tools for self-care and self-analysis of occupational balance. |
| | C19 | Provide space and/or tools for listening, written expression or creative mediation for caregiver overload management. |

c) OCCUPATIONAL ACHIEVEMENT LEVEL

General aims:

- Facilitate autonomy in setting personal goals and meaningful occupational choices.
- Facilitate continuity of learning of critical skills for desired occupational participation and the establishment of new occupational challenges.

Specific aims by stage (summary):

- Facilitate internalisation of habits and execution of significant roles and consolidation of occupational actions that improve occupational performance.
- Optimise the occupational balance of the person and family environment in relation to the new occupations achieved.
- Facilitate information and support for the continuity of performance in the post-intervention phase.

<table>
<tr><th>STAGE AIMS</th><th>INTERV. CORRESP.</th><th>STAGE AIMS SUMMARY</th></tr>
<tr><td>· Facilitate internalisation of habits and execution of roles congruent with the volitional aspects of the person.</td><td>L1</td><td rowspan="5">· Facilitate internalisation of habits and execution of significant roles and consolidation of occupational actions that improve occupational performance.<br>· Optimise the occupational balance of the person and family environment in relation to the new occupations achieved.<br>· Facilitate information and support for the continuity of performance in the post-intervention phase.</td></tr>
<tr><td>· Facilitate information and support for the continuity of performance in the post-intervention phase.</td><td>L4</td></tr>
<tr><td>· Facilitate consolidation of occupational actions that develop and increase performance capabilities.</td><td>L5-9</td></tr>
<tr><td>· Provide support to the family and/or social environment to favour autonomy and independence in the performance of meaningful occupations and roles.</td><td>L10-13</td></tr>
<tr><td>· Optimise the occupational balance of the family environment in relation to the new occupations achieved by the person.</td><td>L11</td></tr>
</table>

| INTERVENTIONS | |
|---|---|
| L1 | Provide positive feedback for the internalisation of habits. |
| L2 | Provide preventive strategies for obstacles to achievement in occupational participation. |
| L3 | Provide strategies for the minimisation and/or management of unavoidable obstacles. |
| L4 | Provide information and support for continued performance in the post-intervention phase. |
| L5 | Provide advice and support in occupational actions to improve motor skills affected by ABI. |
| L6 | Provide advice and support in occupational actions that improve processing skills affected by ABI. |
| L7 | Provide advice and support in occupational actions to improve communication and interaction skills affected by ABI. |
| L8 | Facilitate space and/or tools for listening, written expression or creative mediation for the inscription of a diverse functional narrative of the experienced body. |
| L9 | Support in the emotional management of the occupational impact of ABI and the symptomatology of mental disorder. |
| L10 | Enable the family and/or social environment to recognise occupational milestones and provide subsequent positive feedback to the person. |
| L11 | Facilitate self-analysis strategies of the occupational balance of the social environment and family system. |
| L12 | Provide the social and/or family environment with strategies for the continuity of achievements. |
| L13 | Provide the social and/or family environment with prevention skills in self-care and future care of the family member. |

# REFERENCES

1. Dams-O'Connor K, Landau A, Hoffman J, St De Lore J. Patient perspectives on quality and access to healthcare after brain injury. Brain Inj. 1 de febrero de 2018;32:1-11.

2. Bazarian J, Cernak I, Noble-Haeusslein L, Potolicchio S, Temkin N. Long-term Neurologic Outcomes After Traumatic Brain Injury. J Head Trauma Rehabil. 1 de noviembre de 2009;24:439-51.

3. Whitnall L, Mcmillan T, D Murray G, Teasdale G. Disability in young people and adults after head injury: 5-7 Year follow up of a prospective cohort study. J Neurol Neurosurg Psychiatry. 1 de junio de 2006;77:640-5.

4. Martin-Saez MM, James N. The experience of occupational identity disruption post stroke: a systematic review and meta-ethnography. Disabil Rehabil. 2 de agosto de 2019;1-12.

5. Schwarzbold M, Diaz A, Martins ET, Rufino A, Amante LN, Thais ME, et al. Psychiatric disorders and traumatic brain injury. Neuropsychiatr Dis Treat. agosto de 2008;4(4):797-816.

6. Bergström AL, Eriksson G, Asaba E, Erikson A, Tham K. Complex negotiations: The lived experience of enacting agency after a stroke. Scand J Occup Ther. 2 de enero de 2015;22(1):43-53.

7. Gustavsson M, Guidetti S, Eriksson G, von Koch L, Ytterberg C. Factors affecting outcome in participation one year after stroke: A secondary analysis of a randomized controlled trial. J Rehabil Med. 13 de marzo de 2019;51(3):160-6.

8. Cocks E, Bulsara C, O'Callaghan A, Netto J, Boaden R. Exploring the experiences of people with the dual diagnosis of acquired brain injury and mental illness. Brain Inj. abril de 2014;28(4):414-21.

9. Orlovska S, Pedersen MS, Benros ME, Mortensen PB, Agerbo E, Nordentoft M. Head injury as risk factor for psychiatric disorders: a nationwide register-based follow-up study of 113,906 persons with head injury. Am J Psychiatry. abril de 2014;171(4):463-9.

10. Scholten AC, Haagsma JA, Cnossen MC, Olff M, van Beeck EF, Polinder S. Prevalence of and Risk Factors for Anxiety and Depressive Disorders after Traumatic Brain Injury: A Systematic Review. J Neurotrauma. 2016;33(22):1969-94.

11. Brain Injury Australia, editor. Fact Sheet: Acquired Brain Injury and Mental Illness Services [Internet]. 2007 [citado 1 de julio de 2020]. Disponible en: https://www.braininjuryaustralia.org.au/wp-content/uploads/acquired-brain-injury-and-mental-health-services.pdf

12. The State of Queensland (Queensland Government). Mental Health and ABI | Queensland Health [Internet]. The State of Queensland; [citado 21 de septiembre de 2020]. Disponible en: https://www.health.qld.gov.au/abios/mental-health-and-abi/mental_health

13. Pueblo D del. Informe sobre daño cerebral sobrevenido en España: un acercamiento epidemiológico y sociosanitario (2006) | Defensor del Pueblo [Internet]. [citado 28 de abril de 2021]. Disponible en: https://www.defensordelpueblo.es/informe-

monografico/informe-sobre-dano-cerebral-sobrevenido-en-espana-un-acercamiento-epidemiologico-y-sociosanitario-2006/

14. Fleminger S. Mental Health and Brain Injury factsheet. [Internet]. Headway - Brain injury association; 2016 [citado 1 de marzo de 2019]. Disponible en: https://www.headway.org.uk/media/4051/mental-health-and-brain-injury-factsheet.pdf

15. Sanz-Victoria S. Vivir tras el golpe. Trayectorias sociales de personas que sobreviven a in traumatismo craneoencefálico [Internet] [http://purl.org/dc/dcmitype/Text]. Universitat Autònoma de Barcelona; 2015 [citado 20 de junio de 2019]. Disponible en: https://dialnet.unirioja.es/servlet/tesis?codigo=95811

16. Hellem I, Førland G, Eide K, Ytrehus S. Addressing Uncertainty and Stigma in Social Relations Related to Hidden Dysfunctions Following Acquired Brain Injury. Scand J Disabil Res. 13 de marzo de 2018;20(1):152-61.

17. Marshall S, Bayley M, McCullagh S, Velikonja D, Berrigan L. Clinical practice guidelines for mild traumatic brain injury and persistent symptoms. Can Fam Physician Med Fam Can. marzo de 2012;58(3):257-67, e128-40.

18. World Health Organization. World report on disability [Internet]. 2011 [citado 20 de diciembre de 2020]. Disponible en: https://www.who.int/publications-detail-redirect/9789241564182

19. Shinohara K, Yamada T, Kobayashi N, Forsyth K. The Model of Human Occupation-Based Intervention for Patients with Stroke: A Randomised Trial. Hong Kong J Occup Ther. 1 de diciembre de 2012;22(2):60-9.

20. Kielhofner G. Conceptual Foundations of Occupational Therapy Practice. F.A. Davis Company; 2009. 315 p.

21. de las Heras de Pablo CG. Modelo de Ocupación Humana. Madrid: Editorial Síntesis; 2015.

22. Guidetti S, Eriksson G, Koch L von, Johansson U, Tham K. Activities in Daily Living: The development of a new client-centred ADL intervention for persons with stroke. Scand J Occup Ther. 9 de diciembre de 2020;0(0):1-12.

23. Cotton GS. Occupational Identity Disruption After Traumatic Brain Injury: An Approach to Occupational Therapy Evaluation and Treatment. Occup Ther Health Care [Internet]. 16 de octubre de 2012 [citado 20 de junio de 2019]; Disponible en: https://www.tandfonline.com/doi/pdf/10.3109/07380577.2012.726759

24. Fisher AG. Occupation-centred, occupation-based, occupation-focused: same, same or different? Scand J Occup Ther. mayo de 2013;20(3):162-73.

25. Kielhofner G. Modelo de Ocupación Humana: Teoría y aplicación. 4ª. Buenos Aires: Editorial médica Panamericana; 2011.

26. WHO | WHO Quality of Life-BREF (WHOQOL-BREF) [Internet]. WHO. World Health Organization; [citado 29 de mayo de 2020]. Disponible en: https://www.who.int/substance_abuse/research_tools/whoqolbref/en/

27. Espinoza I, Osorio P, Torrejón MJ, Lucas-Carrasco R, Bunout D. Validación del cuestionario de calidad de vida (WHOQOL-BREF) en adultos mayores chilenos. Rev Médica Chile. mayo de 2011;139(5):579-86.

28. COPM | Canadian Occupational Performance Measure [Internet]. [citado 29 de mayo de 2020]. Disponible en: http://www.thecopm.ca/

29. Eklund M, Argentzell E. Perception of occupational balance by people with mental illness: A new methodology. Scand J Occup Ther. 2016;23(4):304-13.

30. Vidaña-Moya L, Eklund M, Merchán-Baeza JA, Peral-Gómez P, Zango-Martín I, Hultqvist J. Cross-Cultural Adaptation, Validation and Reliability of the Spanish Satisfaction with Daily Occupations-Occupational Balance (SDO-OB): An Evaluation Tool for People with Mental Disorders. Int J Environ Res Public Health. 30 de noviembre de 2020;17(23).

31. Colon H, Haertlein C. Spanish Translation of the Role Checklist. Am J Occup Ther. 1 de septiembre de 2002;56(5):586-9.

32. Alegre-Muelas C, Alegre-Ayala J, Huertas-Hoyas E, Martínez-Piédrola M, Pérez-Corrales J, Máximo-Bocanegra N, et al. Spanish Transcultural Adaptation of the Activity Card Sort. Occup Ther Int [Internet]. 10 de septiembre de 2019 [citado 30 de mayo de 2020];2019. Disponible en: https://www.ncbi.nlm.nih.gov/pmc/articles/PMC6754958/

33. Rintala D, M. Novy D, Garza H, Young M, High W, Chiou-Tan F. Psychometric properties of a Spanish-language version of the Community Integration Questionnaire (CIQ). Rehabil Psychol. 1 de mayo de 2002;47:144-64.

34. Functional Independence Measure (FIM) – Strokengine [Internet]. [citado 25 de mayo de 2021]. Disponible en: https://strokengine.ca/en/assessments/functional-independence-measure-fim/

35. Functional Independence Measure [Internet]. Shirley Ryan AbilityLab. [citado 25 de mayo de 2021]. Disponible en: https://www.sralab.org/rehabilitation-measures/fimr-instrument-fim-fimr-trademark-uniform-data-system-fro-medical
