# Supplementary material for: Study protocol for a non-randomised controlled trial: Community-based occupational therapy intervention on mental health for people with acquired brain injury (COT-MHABI)
Source: PLoS One. 2022 Oct 7;17(10):e0274193. doi: 10.1371/journal.pone.0274193 (PMC9543977; doi:10.1371/journal.pone.0274193)
Supplement: S2 File — (PDF) [file pone.0274193.s004.pdf]

PROPUESTA

Intervención de terapia ocupacional comunitaria en salud mental para personas con daño cerebral adquirido (COT-MHABI)

INTRODUCCIÓN

El daño cerebral adquirido (DCA) se define como aquel daño cerebral posterior al nacimiento, resultado de una fuerza externa (como en el caso del traumatismo craneoencefálico) o debido a procesos no traumáticos (ictus, anoxia, tumores cerebrales, encefalitis, etc.) (1). Las secuelas del DCA moderado-severo abarcan áreas motoras, cognitivas, sensoriales, emocionales y conductuales que afectan a la participación ocupacional, las relaciones sociales y la calidad de vida, produciéndose frecuentemente cambios en la personalidad (2–7).

Este perfil de población muestra una prevalencia significativamente alta de trastorno mental asociado, ya sea por exacerbación de sintomatología preexistente, por la irrupción del daño orgánico, o por la situación psicosocial resultante tras el mismo (8–10). Sin embargo, en el retorno a la comunidad tras la etapa hospitalaria, la atención especializada que reciben es generalmente insuficiente, siendo atendidas desde una perspectiva psiquiátrica y no neurológica, o directamente rechazadas por estos servicios (11–14), principalmente por el desconocimiento de las relaciones entre DCA y salud mental y la invisibilidad de muchos de los déficits asociados (15–17). De esta manera, la persona con DCA moderado-severo se encuentra dentro de la denominación de alta complejidad según la Organización Mundial de la Salud (OMS), ya que manifiesta déficits en funciones corporales, limitación de la actividad y restricción en la participación (18).

Una de las disciplinas más adecuadas para dar soporte a los supervivientes en el manejo de las consecuencias de los déficits producidos por el DCA es la Terapia Ocupacional (19). El Modelo de Ocupación Humana (MOHO), propio de terapia ocupacional, es el modelo focalizado en la ocupación más usado a nivel mundial (19). Es un modelo centrado en la persona y basado en la evidencia, que proporciona evaluaciones e intervenciones específicas focalizadas en la ocupación significativa. Se encuentra fundamentado en tres aspectos principales: la motivación por la ocupación, los hábitos y rutinas, las habilidades de desempeño ocupacional y la influencia del entorno en la participación (19–21). La estructura que proporciona el MOHO nos facilita el acceso a una intervención centrada en la recuperación de ocupaciones significativas para la persona, acompañando la exploración de nuevas opciones de participación, el desempeño de roles y la

reconstrucción activa de una narrativa identitaria interferida por los déficits y su correlato conductual, emocional y social (7,19,20,22,23).

MATERIALES Y MÉTODOS

Objetivos

El objetivo de este estudio se dirige a: (A) diseñar un protocolo de evaluación de la efectividad de una intervención de terapia ocupacional comunitaria basada en el MOHO en salud mental para personas con DCA, desarrollada en entorno domiciliario y focalizada en la ocupación significativa; (B) conocer la contribución a la calidad de vida de la satisfacción con las ocupaciones desempeñadas por un grupo de personas con DCA y trastorno mental.

Criterios de inclusión y exclusión

La población del estudio (grupos experimental y control) estará compuesta por adultos mayores de 18 años con un diagnóstico de DCA medio o grave y un diagnóstico de trastorno neurocognitivo tras el DCA (tal y como se reconoce en la 5ª edición del Manual diagnóstico y estadístico de los trastornos mentales (DSM-5); por definición: evidencia de un deterioro cognitivo significativo con respecto al nivel previo al DCA en uno o más dominios cognitivos (atención, función ejecutiva, aprendizaje y memoria, capacidad perceptivo-motora o cognición social). Puede ocurrir con o sin deterioro del comportamiento (apatía, alteración del estado de ánimo, irritabilidad, desinhibición, síntomas psicóticos, etc.)). Esta población (A) muestra dificultades en la participación laboral con respecto a la situación previa al DCA y presenta necesidades de apoyo, asesoramiento y/o intervención terapéutica específica; (B) se encuentran en situación de alta hospitalaria de unidades de especialización en DCA; (C) están domiciliados en la misma provincia a la que pertenece el hospital proveedor. Además, pueden o no haber sido diagnosticados de un trastorno de salud mental distinto al neurocognitivo, ya sea anterior o posterior al DCA.

Las personas serán excluidas si (A) se encuentran en una situación de desestabilización sintomatológica de deterioro funcional grave que, prioritariamente, requiera un apoyo continuado de unidades especializadas de salud mental, psiquiatría o ingreso sociosanitario; y/o (B) son incapaces de determinar por sí mismas (por razones cognitivas o por elección consciente) cualquier problema en al menos un área ocupacional.

Reclutamiento

La intervención proporcionada al grupo experimental se enmarca dentro del programa de intervención comunitaria de Terapia Ocupacional en salud mental y DCA desarrollado en el Hospital de Neurorrehabilitación Institut Guttmann de Badalona (España). Al tratarse de una intervención muy específica y novedosa, los recursos para su implementación son limitados, generando una lista de espera. Dado que este periodo de espera es superior a la duración de la intervención en el grupo experimental, se invitará a los pacientes en lista de espera a participar en el grupo de control, con la consideración de recibir la intervención en una fase posterior.

Tanto para el grupo experimental como para el de control, la derivación de los pacientes al estudio se realiza a través del servicio de neuropsiquiatría del hospital, que no participa en la intervención, la recogida de datos ni el análisis estadístico de los resultados. Además, los profesionales responsables de la recogida de datos no participarán en la asignación de los sujetos.

Diseño

Se realizará un ensayo clínico controlado no aleatorizado, debido a las razones logísticas expuestas anteriormente. Los puntos de medición de ambos grupos serán comparables, coincidiendo con el sexo, la edad, la zona geográfica de residencia y el diagnóstico. La Fig.1 incluye un diagrama de flujo de las principales partes del estudio.

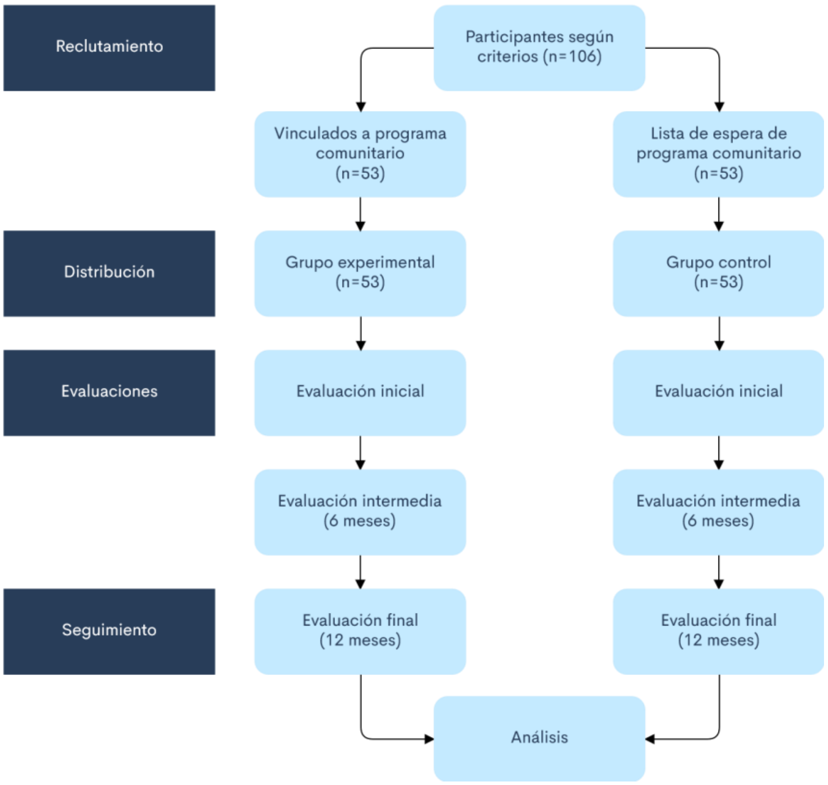

Fig. 1

Intervención

Grupo experimental: intervención de terapia ocupacional comunitaria en salud mental para personas con DCA

Los participantes del grupo experimental recibirán una intervención de terapia ocupacional comunitaria en salud mental, que tendrá en cuenta los problemas relacionados con el DCA, llevada a cabo en un entorno domiciliario y con la ocupación significativa como objetivo principal. El proceso de intervención, en general, seguirá el diagrama mostrado en la Fig. 2.

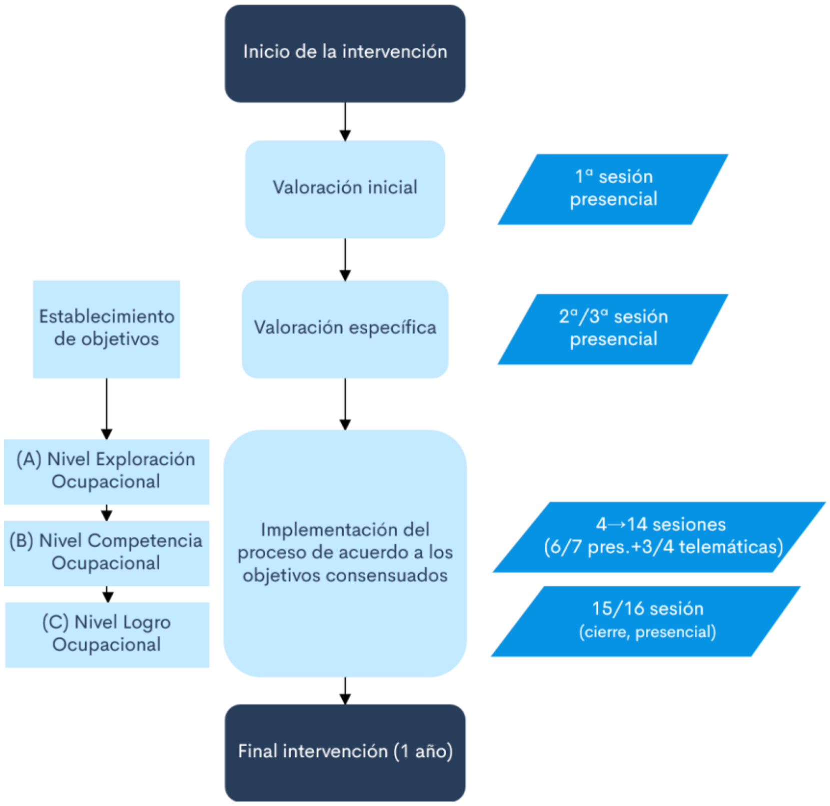

Fig. 2

Una vez que la persona acepte participar en el estudio, se le entregará una hoja informativa y se firmarán los formularios de consentimiento. El investigador principal realizará la intervención y las diferentes evaluaciones del estudio.

Las sesiones de terapia ocupacional se implementarán mediante una intervención basada y focalizada en la ocupación (24) cuya estructura proviene de una adaptación propia del Proceso de Remotivación del Modelo de Ocupación Humana (MOHO) (21,25), que tiene como uno de sus objetivos principales la facilitación de la participación en ocupaciones en personas con dificultades severas de volición (21).

Durante el proceso, de un año de duración, se prevé un máximo de 12 sesiones presenciales y 4 telemáticas sincrónicas en formato de videollamada. Las sesiones presenciales, que transcurrirán en entorno domiciliario y/o comunitario, tendrán una duración de entre 60 y 90 minutos, dependiendo de los objetivos marcados; las telemáticas tendrán una duración aproximada de 45 minutos.

En el caso excepcional en el que no se pueda llevar a cabo la sesión de forma presencial o por videollamada, se realizará vía telefónica. Asimismo, se considera la comunicación puntual tanto telefónica como asincrónica, (mail o mensajes de texto, para posibles intercambios informativos o atención a contingencias, sin que estas comunicaciones constituyan sesión de intervención per se.

Desarrollo de las sesiones

Durante la primera sesión se llevará a cabo la valoración integral inicial, donde el terapeuta ocupacional recogerá información relativa a la historia ocupacional, es decir, actividades significativas para la persona y su impacto en la narrativa vital, y acerca de la participación ocupacional actual, esto es, las ocupaciones que realiza de manera efectiva en el momento de la valoración.

Durante la segunda sesión, se llevará a cabo una valoración específica, donde se realizará un análisis detallado de áreas relevantes y facilitadores/obstaculizadores de la participación ocupacional, junto con una exploración de roles significativos y expectativas de la persona en torno a los mismos. A partir de esta información y de la recogida en la primera sesión, se establecerán los objetivos de manera consensuada con la persona.

Para la consecución de los objetivos, durante el resto del proceso se llevarán a cabo las intervenciones de terapia ocupacional, que estarán definidas a partir de tres niveles secuenciales e interrelacionados que se corresponden con el continuo de cambio definido por el MOHO (21,25). Así, y siguiendo la estructura del anteriormente citado Proceso de Remotivación del modelo, se consideran los siguientes niveles: (A) Exploración ocupacional; (B) Competencia ocupacional; (C) Logro ocupacional. A cada nivel corresponden una serie de etapas compuestas, a su vez, por estrategias de intervención. Un resumen esquemático de la composición de estos niveles se puede ver en la Tabla 1, mientras que el listado completo de las estrategias y objetivos que corresponden a cada nivel aparece en el Apéndice de esta propuesta.

Tabla 1. Composición de los niveles de intervención del proceso

Niveles	Etapas	Objetivos por etapa	Ejemplo de estrategia de intervención
Exploración ocupacional	Validación	Que la persona pueda acceder a experiencias iniciales de capacidad a través de actividad significativa en un entorno seguro.	Acompañamiento y soporte en experiencias iniciales de capacidad en formas ocupacionales asumibles y significativas.
	Disposición a la exploración	Favorecer un estado basal óptimo para permitir la exploración ambiental.	Proporcionar estrategias para favorecer la autonomía en manejo de dificultades relacionadas con el bienestar mental y déficits postDCA.
	Elección	Que la persona pueda aumentar su sentido de capacidad durante la exploración y elección de nuevos hábitos y roles.	Facilitar exploración de nuevas oportunidades volicionales según entorno social y comunitario.
	Eficacia	Que la persona pueda continuar desarrollando su sentido de eficacia a través de la exploración y participación preliminar en hábitos y roles significativos.	Facilitar accesibilidad a formas ocupacionales y tareas propias de las rutinas y roles elegidos.
Competencia ocupacional	Internalización del sentido de la eficacia	Que la persona pueda realizar autoanálisis de capacidad y eficacia en el desempeño, planificando desafíos y objetivos, en congruencia con aspectos de habituación (hábitos y roles) y entorno. Que la persona adquiera estrategias de manejo frente a las dificultades presentes en su desempeño ocupacional.	Proporcionar asesoramiento al entorno para optimizar la capacidad de detección y valoración de hitos de eficacia y su importancia dentro del proceso de rehabilitación.
	Construcción de la narrativa ocupacional	Facilitar realización de acciones ocupacionales y roles que desarrollen y mejoren habilidades afectadas (motoras, de procesamiento y de comunicación e interacción). Facilitar inscripción de la nueva narrativa ocupacional de continuidad (proyecto de rol).	Facilitar la continuidad del proceso de inscripción del sentido de eficacia a través de feedback positivo.
Logro ocupacional	Logro	Facilitar internalización de hábitos y ejecución de roles significativos y consolidación de acciones ocupacionales que mejoren desempeño ocupacional.	Proporcionar estrategias preventivas de obstáculos frente al logro en la participación ocupacional.

		Optimizar el equilibrio ocupacional de la persona y entorno familiar con relación a las nuevas ocupaciones logradas.<br>Facilitar información y soporte para la continuidad del desempeño en la fase posterior a la intervención.

Así, la propuesta general del proceso de intervención será realizar un recorrido progresivo en el que se promoverá que la persona pueda: (A) explorar nuevas ocupaciones mediante el desarrollo de conciencia de la propia capacidad en un entorno seguro; (B) integrar estos nuevos aprendizajes en hábitos congruentes con las exigencias de los roles y entorno, experimentando un aumento de la eficacia personal; (C) llevar a cabo un aumento efectivo de la participación ocupacional que impacte de manera permanente en la identidad ocupacional, en congruencia con el patrón de vida deseado por la persona. Estas intervenciones estarán basadas en un horizonte de búsqueda de la autonomía personal a través del uso de la actividad significativa, la adaptación del entorno físico y la optimización de la relación con el entorno social y familiar (21,25).

El proceso de intervención terapéutico se complementará con acciones de asesoramiento con relación al DCA y salud mental para recursos asistenciales (sociales y sanitarios) y, en general, para agentes comunitarios implicados en la planificación de participación ocupacional de la persona. Asimismo, se realizarán las acciones de conexión de recursos que requiera la intervención, siempre con el objetivo de facilitar una optimización del desempeño ocupacional que facilite la participación y el equilibrio ocupacional, tanto de la persona como de su entorno familiar.

Grupo control: servicios habituales asistenciales (servicios de salud privados/públicos)

Las personas pertenecientes al grupo control recibirán los servicios habituales de sus centros sanitarios de referencia, principalmente acceso a consultas externas y, en algunos casos, tratamientos privados. La temporalidad de estos tratamientos vendrá estimada por los propios centros, en función del estado clínico y/o la demanda del paciente.

Recolección de datos

Las variables que se recogerán en el estudio se evaluarán en tres momentos: evaluación inicial, evaluación intermedia a los seis meses del proceso y evaluación final a los doce meses. Las escalas

utilizadas y su temporalidad serán las mismas tanto para el grupo experimental como para el grupo control. La recogida será llevada a cabo por diferentes investigadores.

Variables principales

Calidad de Vida

Se medirá mediante WHOQoL-BREF (World Health Organization Quality of Life-BREF, version castellano) (26,27). Se trata de un cuestionario autoadministrado genérico creado por el Grupo de estudio de la Calidad de vida de la Organización Mundial de la Salud (OMS). El instrumento tiene 26 preguntas, dos preguntas generales sobre calidad de vida y satisfacción con el estado de salud, y 24 preguntas agrupadas en cuatro áreas: Salud Física, Salud Psicológica, Relaciones Sociales y Ambiente. Puntuaciones mayores indican mejor calidad de vida percibida. Las escalas de respuesta son de tipo Likert de 5 puntos.

Desempeño Ocupacional percibido y satisfacción con el desempeño

Será evaluado mediante COPM (Canadian Occupational Performance Measure, versión castellano) (21,28), medida autoadministrada propia de terapia ocupacional basada en la client-centred practice, que ayuda a establecer las necesidades ocupacionales. Evalúa los cambios en la autopercepción del desempeño ocupacional y de la satisfacción con ese desempeño tras la intervención realizada.

Variables secundarias

Satisfacción con las ocupaciones y equilibrio ocupacional

Se medirá mediante la escala SOD-EO (Satisfaction with Daily Occupation and Occupational Balance, SDO-OB, versión castellano) (29,30). El instrumento evalúa la satisfacción en trece áreas ocupacionales, organizadas en cuatro dominios (productividad, ocio, tareas del hogar y autocuidado) en términos de nivel de actividad de la persona, satisfacción y equilibrio ocupacionales. Las puntuaciones del nivel de satisfacción se responden en base a una escala de 7 ítems.

Participación y satisfacción con los roles desempeñados

Se evaluarán mediante la RCv3 (Role Checklist Version 3: Participation and Satisfaction) (31). El instrumento evalúa la percepción del desempeño de roles de la persona, la satisfacción con los mismos y el deseo de participar en el futuro en otros roles.

Nivel de participación autopercibido en actividades

Se evaluará a partir de la ACS (Activity Card Sort, versión en español) (32). Se trata de un instrumento compuesto por un total de 89 fotografías, utilizado en terapia ocupacional para realizar una exploración conjunta con la persona, anotando un catálogo de actividades realizadas en todas las áreas, tanto previa como posteriormente a la lesión o situación que interfiere en el desempeño. Es útil para identificar pérdida de participación, establecer objetivos y analizar evolución. Los ítems incluyen 20 actividades instrumentales, 17 actividades sociales y 35 actividades de ocio de baja demanda física y 17 de alta demanda.

Integración comunitaria

Se medirá a partir de los resultados recogidos en la CIQ (Community Integration Questionnaire) (33). Valora las limitaciones en la ejecución de los roles sociales y la interacción en comunidad de personas con DCA. Presenta tres dimensiones principales: integración doméstica, integración social y productividad. El rango de puntación total se encuentra entre 0-29 y la mayoría de ítems tienen un rango de 0-2. Valores más altos representan mayor integración en la comunidad e independencia.

Independencia funcional

Se evaluará desde la FIM (Functional Independence Measure) (34,35). La escala representa un sistema uniforme de medida para la discapacidad basada en la International Classification of Impairment, Disabilities and Handicaps. Valora seis áreas funcionales (autocuidado, control de esfínteres, transferencias, locomoción, comunicación y cognición social) dentro de dos dominios (motor y cognitivo). Cada ítem es puntuado en una escala tipo Likert de 7 puntos. Los ítems se basan más en el desempeño que en la capacidad y se registran de manera heteroadministrada.

Satisfacción con el proceso de intervención

Se recogerá de manera cualitativa y cuantitativa mediante cuestionarios de satisfacción propios. En este caso, se trata de un cuestionario para la persona participante en el proceso y otro para el principal familiar referente, si lo hubiere. Ambos cuestionarios están compuestos de siete preguntas acerca de la percepción subjetiva de los resultados obtenidos tras la intervención con escala de respuesta tipo Likert de 7 puntos. Por otro lado, hay 4 preguntas más que, con una escala del 1 al 10, pretenden recoger la opinión de la calidad de la intervención. Por último, se incluirá un apartado donde, de manera cualitativa, la persona puede expresar otros comentarios no recogidos en las preguntas anteriores.

CRONOGRAMA

Tabla 3. Cronograma

			Fechas				
Tareas	Referencia institucional	Investigadores	2019	2020	2021	2022	2023
DISEÑO							
Concepción idea y diseño estudio	UVic-IG	MARR - BCM-JAMB	X				
Planificación	UVic-IG	MARR - BCM-JAMB	X	X	X		
Redefinición diseño	UVic-IG	MARR - BCM-JAMB		X	X		
INTERVENCIÓN: RECOGIDA Y SEGUIMIENTO							
Recogida datos clínicos	UVic-IG	MARR - BCM-JAMB		X	X	X	
ANÁLISIS							
Procesamiento de datos	UVic-IG	MARR - BCM-JAMB				X	
Análisis estadístico	UVic-IG	MARR - BCM-JAMB				X	
DISEMINACIÓN							
Jornadas, conferencias	IG	MARR - BCM-JAMB			X	X	X
Artículos científicos	UVic-IG	MARR - BCM-JAMB			X	X	X

UVic: Universitat de Vic – Universitat Central de Catalunya; IG: Institut Guttmann

MARR: Marco Antonio Raya-Ruiz; BCM: Beatriz Castaño-Monsalve; JAMB: Jose Antonio Merchán-Baeza

APPENDIX

1. DESCRIPCIÓN DE NIVELES, ETAPAS E INTERVENCIONES DE PROCESO

a) NIVEL EXPLORACIÓN OCUPACIONAL

Objetivos generales:

- Favorecer establecimiento del vínculo terapéutico.
- Que la persona pueda participar en actividades significativas posteriores al DCA (reconexión previas/exploración nuevas) en su entorno domiciliario y comunitario.
- Que la persona pueda acceder a nuevas oportunidades de experimentación y elección de posibilidades ocupacionales congruentes con volición, capacidad de desempeño y entorno.
- Fomentar volición remanente y favorecer optimización en la exploración ambiental.
- Que la persona pueda acceder a experiencias iniciales de participación grupal (social y familiar) en ambiente seguro.

Objetivos específicos por etapa (síntesis):

- VALIDACIÓN: Que la persona pueda acceder a experiencias iniciales de capacidad mediante el uso de actividad significativa en un entorno seguro.
- DISPOSICIÓN A LA EXPLORACIÓN: Favorecer un estado basal óptimo para permitir la exploración ambiental.
- ELECCIÓN: Que la persona pueda aumentar su sentido de capacidad durante la exploración y elección de nuevos hábitos y roles.
- EFICACIA: Que la persona pueda continuar desarrollando su sentido de eficacia a través de la exploración y participación preliminar en hábitos y roles significativos.

OBJETIVOS DE ETAPA: VALIDACIÓN	CORRESPONDENCIA INTERVENCIÓN	SÍNTESIS OBJETIVOS ETAPA
· Que la persona pueda acceder a **experiencias iniciales de capacidad** mediante el uso de actividad significativa.	E2-4	Que la persona pueda acceder a experiencias iniciales de capacidad mediante el uso de actividad significativa en un entorno seguro.
· Establecer un **espacio de mediación y comunicación entre familia y persona**.	E5	
· Facilitar **seguridad** en el entorno (social, familiar y físico) para la promoción de la exploración ocupacional.	E5-6	
OBJETIVOS DE ETAPA: DISPOSICIÓN A LA EXPLORACIÓN		
· Favorecer un **estado basal óptimo para permitir la exploración** ambiental.	E7-11	Favorecer un estado basal óptimo para permitir la exploración ambiental.
· Proporcionar soporte al **entorno** social para facilitar exploración segura.	E12	
OBJETIVOS DE ETAPA: ELECCIÓN		
. Que la persona pueda **aumentar su sentido de capacidad** a partir de la emergencia de la autovalidación.	E13-15	Que la persona pueda acceder a experiencias iniciales de capacidad mediante el uso de actividad significativa en un entorno seguro.
· Facilitar la **exploración y elección de nuevos hábitos y roles**.	E16,17	
· Facilitar **soporte del entorno social y familiar a las elecciones** realizadas por la persona.	E18	
OBJETIVOS DE ETAPA: EFICACIA		
· Facilitar participación preliminar en nuevos hábitos y roles elegidos.	E19,20	Facilitar que la persona pueda continuar desarrollando su sentido de eficacia a través de la exploración y participación preliminar en hábitos y roles significativos.
· Facilitar reconstrucción consciente de la **identidad ocupacional a través de la autovalidación.**	E21-22	
· Favorecer **respuesta positiva** del entorno para asegurar sentido de la eficacia y continuidad en la exploración.	E23-24	

ETAPAS	INTERVENCIONES	
VALIDACIÓN	E1	Análisis inicial de aspectos volitivos, de habituación, componentes objetivos (HH motoras, procesamiento, comunicación y sensoriales) y subjetivos (cuerpo vivido).
	E2	Acompañamiento y soporte en experiencias iniciales de capacidad en formas ocupacionales asumibles y significativas.
	E3	Soporte emocional en la expresión de las dificultades y esfuerzos con relación al cuerpo vivido y las habilidades de desempeño.
	E4	Asesoramiento a la persona en el manejo de aspectos propios del DCA y SM con relación a la participación ocupacional.
	E5	Facilitar expresión de demandas en espacios de interacción familiar y/o social.
	E6	Proporcionar al ambiente social y familiar asesoramiento en aspectos y dificultades propias del DCA y SM.
DISPOSICIÓN A EXPLORAR AMBIENTE	E7	Soporte físico y/o emocional a la exploración de nuevas acciones ocupacionales, espacios, objetos y relaciones.
	E8	Asesoramiento y entrenamiento en el uso de productos de soporte.
	E9	Asesoramiento y entrenamiento en gestión autónoma de la toma de medicación.
	E10	Proporcionar estrategias para favorecer la autonomía en manejo de dificultades relacionadas con el bienestar mental y secuelas postDCA.
	E11	Asesoramiento y soporte para favorecer autonomía en manejo de dificultades relacionadas con el consumo de tóxicos.
	E12	Proporcionar asesoramiento y espacio de diálogo ambiente-persona.
ELECCIÓN	E13	Proporcionar estrategias de autoanálisis y autovalidación de aspectos volitivos presentes.
	E14	Acompañar en la toma de decisiones con relación a los aspectos volitivos significativos.
	E15	Facilitar exploración de nuevas oportunidades volicionales según entorno social y comunitario.
	E16	Facilitar estrategias para el establecimiento de nuevas rutinas.
	E17	Facilitar incremento tiempo empleado y frecuencia de rutinas significativas.
	E18	Proporcionar al entorno familiar estrategias para facilitar su adaptación a elecciones significativas de la persona.
PLACER Y EFICACIA EN LA ACCIÓN	E19	Facilitar accesibilidad a formas ocupacionales y tareas propias de las rutinas y roles elegidos.
	E20	Proporcionar estrategias para aumentar participación en rutinas diarias y semanales.
	E21	Facilitar la emergencia del proceso de inscripción del sentido de eficacia a través de feedback positivo.
	E22	Facilitar mediante feedback el autoanálisis de capacidades de desempeño.
	E23	Proporcionar feedback al entorno sobre avances conseguidos por la persona en las diferentes etapas del nivel de exploración.
	E24	Proporcionar estrategias al entorno para aumentar eficacia en el desempeño de la persona.

b) NIVEL COMPETENCIA OCUPACIONAL

Objetivos generales:

- Que la persona pueda planificar y ejecutar desafíos (congruentes con desempeño y entorno) en su participación ocupacional para aumentar el sentido de eficacia, con el soporte del entorno.
- Que la persona pueda incrementar expectativas de capacidad en el desempeño para facilitar desarrollo de HH.
- Acompañamiento a la persona en el proceso de construcción de su narrativa ocupacional, reforzando procesos de constitución de nueva identidad ocupacional deseada.

Objetivos específicos por etapa (síntesis):

- INTERNALIZACIÓN SENTIDO EFICACIA: Que la persona pueda realizar autoanálisis de capacidad y eficacia en el desempeño, planificando desafíos y objetivos, en congruencia con aspectos de habituación (hábitos y roles) y entorno. Que la persona adquiera estrategias de manejo frente a las dificultades presentes en su desempeño ocupacional.
- CONSTRUCCIÓN NARRATIVA OCUPACIONAL: Facilitar realización de acciones ocupacionales y roles que desarrollen y mejoren habilidades (motoras, de procesamiento y de comunicación e interacción) afectadas por el DCA. Facilitar al entorno autonomía en el cuidado y acompañamiento a la evolución de la competencia ocupacional. Facilitar inscripción de la nueva narrativa ocupacional de continuidad (proyecto de rol).

OBJETIVOS DE ETAPA: INTERNAL.SENTIDO EFICACIA	CORRESPONDENCIA INTERVENCIÓN	SÍNTESIS OBJETIVOS ETAPA
· Facilitar el acceso a oportunidades de desafío ocupacional según congruencia con aspectos volicionales, roles y capacidad de desempeño.	C1	Que la persona pueda realizar autoanálisis de capacidad y eficacia en el desempeño, planificando desafíos y objetivos, en congruencia con aspectos de habituación (hábitos y roles) y entorno. Que la persona adquiera estrategias de manejo frente a las dificultades presentes en su desempeño ocupacional.
· Que la persona pueda llevar a cabo el planteamiento de nuevas metas y objetivos de manera autónoma.	C2	
· Favorecer el establecimiento de rutinas y hábitos con relación a roles deseados y capacidad de desempeño.	C3,4	
· Facilitar emergencia de estrategias de manejo en el desempeño.	C5,6	
· Facilitar soporte del entorno familiar y social para favorecer el aumento de sentido de eficacia y apoyar el desempeño de roles.	C7-9	
OBJETIVOS DE ETAPA: CONSTRUCCIÓN DE LA NARRATIVA OCUPACIONAL		
· Facilitar la continuidad autónoma de acciones ocupacionales significativas.	C10-13	Facilitar realización de acciones ocupacionales y roles que desarrollen y mejoren habilidades (motoras, de procesamiento y de comunicación e interacción) afectadas por el DCA. Facilitar al entorno autonomía en el cuidado y acompañamiento a la evolución de la competencia ocupacional. Facilitar inscripción de la nueva narrativa ocupacional de continuidad (proyecto de rol).
· Facilitar realización de acciones ocupacionales y roles que desarrollen y mejoren habilidades (motoras, de procesamiento y de comunicación e interacción) afectadas por el DCA y necesarias para su participación ocupacional.	C14-15	
· Proporcionar soporte en la expresión del cuerpo vivido y su lugar en el continuo ocupacional.	C17	
· Facilitar la inscripción de una nueva narrativa de continuidad (proyecto de rol) con relación al desempeño ocupacional.	C17	
· Aumentar capacidad de gestión de sobrecarga en los familiares.	C18,19	
· Proporcionar soporte en la expresión del impacto del DCA y su inscripción en el continuo ocupacional dentro de la historia de vida del sistema familiar.	C19	

ETAPAS	INTERVENCIONES	
INTERNALIZACIÓN SENTIDO EFICACIA	C1	Soporte y acompañamiento en la gradación y experimentación de AVD y ocio para facilitar desafío ocupacional.
	C2	Acompañamiento en la actualización autónoma de los objetivos del proceso.
	C3	Facilitar estructuración espacio-temporal de las tareas y ocupaciones diarias.
	C4	Facilitar autoanálisis de eficacia en el desempeño de rutinas y hábitos.
	C5	Facilitar optimización del uso de fortalezas objetivas y subjetivas en el desempeño ocupacional.
	C6	Asesoramiento en estrategias de manejo frente a dificultades en el desempeño.
	C7	Facilitar en el entorno la comprensión de las fortalezas y debilidades de la persona con relación a su desempeño.
	C8	Facilitar al entorno la capacidad de proporcionar feedback positivo a la persona sobre su desempeño ocupacional.
	C9	Proporcionar asesoramiento al entorno para optimizar la capacidad de detección y valoración de hitos de eficacia y su importancia dentro del proceso de rehabilitación.
CONSTRUCCIÓN NARRATIVA OCUPACIONAL	C10	Facilitar el autoanálisis de hitos volicionales.
	C11	Facilitar la continuidad del proceso de inscripción del sentido de eficacia a través de feedback positivo.
	C12	Facilitar autoanálisis de desempeño eficaz/no eficaz.
	C13	Facilitar estrategias de resolución y manejo frente a acciones de desempeño ocupacional no exitosas.
	C14	Asesoramiento y soporte a la realización de acciones ocupacionales que desarrollen y mejoren habilidades motoras afectadas por el DCA.
	C15	Asesoramiento y soporte a la realización de acciones ocupacionales que desarrollen y mejoren habilidades de procesamiento afectadas por el DCA.
	C16	Asesoramiento y soporte a la realización de acciones ocupacionales que desarrollen y mejoren habilidades de comunicación e interacción afectadas por el DCA.
	C17	Facilitar espacio y/o herramientas de escucha, expresión escrita o mediación creativa para autoreconocimiento de proceso.
	C18	Proporcionar al ambiente social/familiar herramientas de autocuidado y autoanálisis de equilibrio ocupacional.
	C19	Facilitar espacio y/o herramientas de escucha, expresión escrita o mediación creativa para gestión sobrecarga cuidadores.

c) NIVEL LOGRO OCUPACIONAL

Objetivos generales:

- Facilitar autonomía en el establecimiento de objetivos personales y elecciones ocupacionales significativas.
- Facilitar continuidad de aprendizaje de HH críticas para la participación ocupacional deseada y el establecimiento de nuevos desafíos ocupacionales.

Objetivos específicos por etapa (síntesis):

- Facilitar internalización de hábitos y ejecución de roles significativos y consolidación de acciones ocupacionales que mejoren desempeño ocupacional.
- Optimizar el equilibrio ocupacional de la persona y entorno familiar con relación a las nuevas ocupaciones logradas.
- Facilitar información y soporte para la continuidad del desempeño en la fase posterior a la intervención.

OBJETIVOS ETAPA	CORRESPOND. INTERVENCIÓN	SÍNTESIS POR ETAPA
· Facilitar internalización de hábitos y ejecución de roles congruentes con los aspectos volitivos de la persona.	L1	· Facilitar internalización de hábitos y ejecución de roles significativos y consolidación de acciones ocupacionales que mejoren desempeño ocupacional.<br>· Optimizar el equilibrio ocupacional de la persona y entorno familiar con relación a las nuevas ocupaciones logradas.<br>· Facilitar información y soporte para la continuidad del desempeño en la fase posterior a la intervención.
· Facilitar información y soporte para la continuidad del desempeño en la fase posterior a la intervención.	L4	
· Facilitar consolidación de acciones ocupacionales que desarrollen y aumenten capacidades de desempeño.	L5-9	
· Proporcionar soporte al entorno para favorecer la autonomía e independencia en desempeño de ocupaciones y roles significativos.	L10-13	
· Optimizar el equilibrio ocupacional del entorno familiar con relación a las nuevas ocupaciones logradas por la persona.	L11	

INTERVENCIONES	
L1	Proporcionar feedback positivo para la internalización de hábitos.
L2	Proporcionar estrategias preventivas de obstáculos frente al logro en la participación ocupacional.
L3	Proporcionar estrategias de minimización y/o gestión de obstáculos ineludibles.
L4	Facilitar información y soporte para la continuidad del desempeño en la fase posterior a la intervención.
L5	Asesoramiento y soporte en acciones ocupacionales que mejoren habilidades motoras afectadas por el DCA.
L6	Asesoramiento y soporte en acciones ocupacionales que mejoren habilidades de procesamiento afectadas por el DCA.
L7	Asesoramiento y soporte en acciones ocupacionales que mejoren habilidades de comunicación e interacción afectadas por el DCA.
L8	Facilitar espacio y/o herramientas de escucha, expresión escrita o mediación creativa para inscripción de una narrativa funcional diversa del cuerpo vivido.
L9	Soporte en la gestión emocional del impacto ocupacional del DCA y la sintomatología propia de trastorno mental.
L10	Facilitar al entorno familiar la capacidad de reconocimiento de hitos ocupacionales y posterior feedback positivo a la persona.
L11	Facilitar estrategias de autoanálisis del equilibrio ocupacional del ambiente y sistema familiar.
L12	Proporcionar al entorno estrategias de continuidad de los logros conseguidos.
L13	Facilitar al ambiente social y familiar habilidades de prevención en autocuidados y cuidado futuro del familiar.

BIBLIOGRAFÍA

1. Dams-O'Connor K, Landau A, Hoffman J, St De Lore J. Patient perspectives on quality and access to healthcare after brain injury. Brain Inj. 1 de febrero de 2018;32:1-11.

2. Bazarian J, Cernak I, Noble-Haeusslein L, Potolicchio S, Temkin N. Long-term Neurologic Outcomes After Traumatic Brain Injury. J Head Trauma Rehabil. 1 de noviembre de 2009;24:439-51.

3. Whitnall L, Mcmillan T, D Murray G, Teasdale G. Disability in young people and adults after head injury: 5-7 Year follow up of a prospective cohort study. J Neurol Neurosurg Psychiatry. 1 de junio de 2006;77:640-5.

4. Martin-Saez MM, James N. The experience of occupational identity disruption post stroke: a systematic review and meta-ethnography. Disabil Rehabil. 2 de agosto de 2019;1-12.

5. Schwarzbold M, Diaz A, Martins ET, Rufino A, Amante LN, Thais ME, et al. Psychiatric disorders and traumatic brain injury. Neuropsychiatr Dis Treat. agosto de 2008;4(4):797-816.

6. Bergström AL, Eriksson G, Asaba E, Erikson A, Tham K. Complex negotiations: The lived experience of enacting agency after a stroke. Scand J Occup Ther. 2 de enero de 2015;22(1):43-53.

7. Gustavsson M, Guidetti S, Eriksson G, von Koch L, Ytterberg C. Factors affecting outcome in participation one year after stroke: A secondary analysis of a randomized controlled trial. J Rehabil Med. 13 de marzo de 2019;51(3):160-6.

8. Cocks E, Bulsara C, O'Callaghan A, Netto J, Boaden R. Exploring the experiences of people with the dual diagnosis of acquired brain injury and mental illness. Brain Inj. abril de 2014;28(4):414-21.

9. Orlovska S, Pedersen MS, Benros ME, Mortensen PB, Agerbo E, Nordentoft M. Head injury as risk factor for psychiatric disorders: a nationwide register-based follow-up study of 113,906 persons with head injury. Am J Psychiatry. abril de 2014;171(4):463-9.

10. Scholten AC, Haagsma JA, Cnossen MC, Olff M, van Beeck EF, Polinder S. Prevalence of and Risk Factors for Anxiety and Depressive Disorders after Traumatic Brain Injury: A Systematic Review. J Neurotrauma. 2016;33(22):1969-94.

11. Brain Injury Australia, editor. Fact Sheet: Acquired Brain Injury and Mental Illness Services [Internet]. 2007 [citado 1 de julio de 2020]. Disponible en: https://www.braininjuryaustralia.org.au/wp-content/uploads/acquired-brain-injury-and-mental-health-services.pdf

12. The State of Queensland (Queensland Government). Mental Health and ABI | Queensland Health [Internet]. The State of Queensland; [citado 21 de septiembre de 2020]. Disponible en: https://www.health.qld.gov.au/abios/mental-health-and-abi/mental_health

13. Pueblo D del. Informe sobre daño cerebral sobrevenido en España: un acercamiento epidemiológico y sociosanitario (2006) | Defensor del Pueblo [Internet]. [citado 28 de abril de 2021]. Disponible en: https://www.defensordelpueblo.es/informe-

monografico/informe-sobre-dano-cerebral-sobrevenido-en-espana-un-acercamiento-epidemiologico-y-sociosanitario-2006/

14. Fleminger S. Mental Health and Brain Injury factsheet. [Internet]. Headway - Brain injury association; 2016 [citado 1 de marzo de 2019]. Disponible en: https://www.headway.org.uk/media/4051/mental-health-and-brain-injury-factsheet.pdf

15. Sanz-Victoria S. Vivir tras el golpe. Trayectorias sociales de personas que sobreviven a in traumatismo craneoencefálico [Internet] [http://purl.org/dc/dcmitype/Text]. Universitat Autònoma de Barcelona; 2015 [citado 20 de junio de 2019]. Disponible en: https://dialnet.unirioja.es/servlet/tesis?codigo=95811

16. Hellem I, Førland G, Eide K, Ytrehus S. Addressing Uncertainty and Stigma in Social Relations Related to Hidden Dysfunctions Following Acquired Brain Injury. Scand J Disabil Res. 13 de marzo de 2018;20(1):152-61.

17. Marshall S, Bayley M, McCullagh S, Velikonja D, Berrigan L. Clinical practice guidelines for mild traumatic brain injury and persistent symptoms. Can Fam Physician Med Fam Can. marzo de 2012;58(3):257-67, e128-40.

18. World Health Organization. World report on disability [Internet]. 2011 [citado 20 de diciembre de 2020]. Disponible en: https://www.who.int/publications-detail-redirect/9789241564182

19. Shinohara K, Yamada T, Kobayashi N, Forsyth K. The Model of Human Occupation-Based Intervention for Patients with Stroke: A Randomised Trial. Hong Kong J Occup Ther. 1 de diciembre de 2012;22(2):60-9.

20. Kielhofner G. Conceptual Foundations of Occupational Therapy Practice. F.A. Davis Company; 2009. 315 p.

21. de las Heras de Pablo CG. Modelo de Ocupación Humana. Madrid: Editorial Síntesis; 2015.

22. Guidetti S, Eriksson G, Koch L von, Johansson U, Tham K. Activities in Daily Living: The development of a new client-centred ADL intervention for persons with stroke. Scand J Occup Ther. 9 de diciembre de 2020;0(0):1-12.

23. Cotton GS. Occupational Identity Disruption After Traumatic Brain Injury: An Approach to Occupational Therapy Evaluation and Treatment. Occup Ther Health Care [Internet]. 16 de octubre de 2012 [citado 20 de junio de 2019]; Disponible en: https://www.tandfonline.com/doi/pdf/10.3109/07380577.2012.726759

24. Fisher AG. Occupation-centred, occupation-based, occupation-focused: same, same or different? Scand J Occup Ther. mayo de 2013;20(3):162-73.

25. Kielhofner G. Modelo de Ocupación Humana: Teoría y aplicación. 4ª. Buenos Aires: Editorial médica Panamericana; 2011.

26. WHO | WHO Quality of Life-BREF (WHOQOL-BREF) [Internet]. WHO. World Health Organization; [citado 29 de mayo de 2020]. Disponible en: https://www.who.int/substance_abuse/research_tools/whoqolbref/en/

27. Espinoza I, Osorio P, Torrejón MJ, Lucas-Carrasco R, Bunout D. Validación del cuestionario de calidad de vida (WHOQOL-BREF) en adultos mayores chilenos. Rev Médica Chile. mayo de 2011;139(5):579-86.

28. COPM | Canadian Occupational Performance Measure [Internet]. [citado 29 de mayo de 2020]. Disponible en: http://www.thecopm.ca/

29. Eklund M, Argentzell E. Perception of occupational balance by people with mental illness: A new methodology. Scand J Occup Ther. 2016;23(4):304-13.

30. Vidaña-Moya L, Eklund M, Merchán-Baeza JA, Peral-Gómez P, Zango-Martín I, Hultqvist J. Cross-Cultural Adaptation, Validation and Reliability of the Spanish Satisfaction with Daily Occupations-Occupational Balance (SDO-OB): An Evaluation Tool for People with Mental Disorders. Int J Environ Res Public Health. 30 de noviembre de 2020;17(23).

31. Colon H, Haertlein C. Spanish Translation of the Role Checklist. Am J Occup Ther. 1 de septiembre de 2002;56(5):586-9.

32. Alegre-Muelas C, Alegre-Ayala J, Huertas-Hoyas E, Martínez-Piédrola M, Pérez-Corrales J, Máximo-Bocanegra N, et al. Spanish Transcultural Adaptation of the Activity Card Sort. Occup Ther Int [Internet]. 10 de septiembre de 2019 [citado 30 de mayo de 2020];2019. Disponible en: https://www.ncbi.nlm.nih.gov/pmc/articles/PMC6754958/

33. Rintala D, M. Novy D, Garza H, Young M, High W, Chiou-Tan F. Psychometric properties of a Spanish-language version of the Community Integration Questionnaire (CIQ). Rehabil Psychol. 1 de mayo de 2002;47:144-64.

34. Functional Independence Measure (FIM) – Strokengine [Internet]. [citado 25 de mayo de 2021]. Disponible en: https://strokengine.ca/en/assessments/functional-independence-measure-fim/

35. Functional Independence Measure [Internet]. Shirley Ryan AbilityLab. [citado 25 de mayo de 2021]. Disponible en: https://www.sralab.org/rehabilitation-measures/fimr-instrument-fim-fimr-trademark-uniform-data-system-fro-medical
