# Supplementary material for: Study protocol for a non-randomised controlled trial: Community-based occupational therapy intervention on mental health for people with acquired brain injury (COT-MHABI)
Source: PLoS One. 2022 Oct 7;17(10):e0274193. doi: 10.1371/journal.pone.0274193 (PMC9543977; doi:10.1371/journal.pone.0274193)
Supplement: Attachment — Submitted filename: Response to reviewers.docx [file pone.0274193.s005.docx]

RESPONSE TO REVIEWERS - PONE-D-21-30716

Note: author uses red for responses under the original comment

1. Does the manuscript provide a valid rationale for the proposed study, with clearly identified and justified research questions?

The research question outlined is expected to address a valid academic problem or topic and contribute to the base of knowledge in the field.

Reviewer #1: Partly

2. Is the protocol technically sound and planned in a manner that will lead to a meaningful outcome and allow testing the stated hypotheses?

The manuscript should describe the methods in sufficient detail to prevent undisclosed flexibility in the experimental procedure or analysis pipeline, including sufficient outcome-neutral conditions (e.g. necessary controls, absence of floor or ceiling effects) to test the proposed hypotheses and a statistical power analysis where applicable. As there may be aspects of the methodology and analysis which can only be refined once the work is undertaken, authors should outline potential assumptions and explicitly describe what aspects of the proposed analyses, if any, are exploratory.

Reviewer #1: Partly

3. Is the methodology feasible and described in sufficient detail to allow the work to be replicable?


Descriptions of methods and materials in the protocol should be reported in sufficient detail for another researcher to reproduce all experiments and analyses. The protocol should describe the appropriate controls, sample size calculations, and replication needed to ensure that the data are robust and reproducible.

Reviewer #1: No

Response to question 1, 2 and 3: Since some time has passed since our manuscript proposal and, after reading the reviewer's comments, we have revised most of the sections to bring them more in line with what was suggested. In this regard, we expect this sections to be considerably improved. Thank you for the comments.

4. Have the authors described where all data underlying the findings will be made available when the study is complete?

The [PLOS Data policy](https://eur02.safelinks.protection.outlook.com/?url=https%3A%2F%2Fjournals.plos.org%2Fplosone%2Fs%2Fmaterials-and-software-sharing&data=05%7C01%7Cjosan.merchan%40uvic.cat%7Cc30cf119e48e4d571e1808da4b451c74%7C05bec0394f4f40879f2e1e71aa64c56e%7C0%7C0%7C637905057446275951%7CUnknown%7CTWFpbGZsb3d8eyJWIjoiMC4wLjAwMDAiLCJQIjoiV2luMzIiLCJBTiI6Ik1haWwiLCJXVCI6Mn0%3D%7C3000%7C%7C%7C&sdata=A%2BpGJwf3Ekj%2BjzgjOsv1WckOY5bHh3yLq5hoSOsmL%2F4%3D&reserved=0) requires authors to make all data underlying the findings described in their manuscript fully available without restriction, with rare exception, at the time of publication. The data should be provided as part of the manuscript or its supporting information, or deposited to a public repository. For example, in addition to summary statistics, the data points behind means, medians and variance measures should be available. If there are restrictions on publicly sharing data—e.g. participant privacy or use of data from a third party—those must be specified.

Reviewer #1: Yes

5. Is the manuscript presented in an intelligible fashion and written in standard English?

PLOS ONE does not copyedit accepted manuscripts, so the language in submitted articles must be clear, correct, and unambiguous. Any typographical or grammatical errors should be corrected at revision, so please note any specific errors here.

Reviewer #1: Yes

6. Review Comments to the Author

Please use the space provided to explain your answers to the questions above and, if applicable, provide comments about issues authors must address before this protocol can be accepted for publication. You may also include additional comments for the author, including concerns about research or publication ethics.

You may also provide optional suggestions and comments to authors that they might find helpful in planning their study.

(Please upload your review as an attachment if it exceeds 20,000 characters)

Reviewer #1: The manuscript entitled ‘Community-based occupational therapy intervention on mental health for people with acquired brain injury (COT-MHABI): Study protocol for non-randomized controlled Trial’

The manuscript requires further improvement based on the following comments.

The title requires revision e.g. to begin with ‘Study protocol for a non-randomized controlled trial……..

Response: Thank you for the suggestion. Change has been made

Abstract
Page 2, the sentence ‘intervention based on MOHO and developed in a home setting’ requires revision. The comparative group is to be clearly stated. The abstract requires English editing. Response: Thank you for the suggestion. Changes have been made and, in general, an official translation service (PRS) has been used.

Materials and methods

Page 6, typo etc.)). Response: Fixed, thank you.

Page 6, the numbering item A, B, C could be replaced with, (i), (ii), (iii) or (a), (b), (c). This applies to other sections of the manuscript. Response: Changes had been made plus best definition of inclusion/exclusion criteria

Recruitment of subjects

Page 7, more information on how the subjects were allocated to intervention and control groups, who allocate/placed the subjects into the groups and collect the data to be clearly stated. Page 7, how long is the estimated waiting list in the control group to be clearly stated. Response: Thanks for the comments, we have improved the definition of this section and we hope that it will further clarify the procedure.

Page 9, the objectives to be placed after introduction before materials and methods section. The objectives require English editing. ‘developed in a home setting’ not clear. The words 'meaningful occupation' and ‘ to know the contribution’ are to be revised. Meaningful occupation to be replaced with meaningful activities. Response: Thanks for the suggestion. We have changed the concept of home-setting to community-based, for being more complete. We have also corrected the phrasing of the objectives. Regarding the change from meaningful occupation to meaningful activity, we agree with your suggestion, also in line with articles such as Eakman (2013).

Page 8 Figure 1, more information/important point(s) on what was received by intervention and control group to be clearly stated in the figure. Response: on the basis of your suggestion, we have changed the figure to add this issue.

Intervention
Page 11, the sentence ‘which has as one of its main objectives the facilitation of participation in occupations in people with severe volitional difficulties’ to be revised. Response: revision done, thanks for the comment.

All inventories/questionnaires whether all are Spanish versions to be indicated. Response: changes have been made.

Sample size calculation

One or two-tailed test and the attrition rate to be stated. Beta=0.20 and Power=1-0.20=0.80. Response: thank you very much for the comment, we have made changes in this aspect, adding the consideration of the two tails, also in the calculation of the power size.

Statistical analysis

A write-up on possible missing data and methods of handling missing data is to be added. Response: thank you so much for the suggestion. We have added a clarification in this respect, using the multiple imputation method.

Page 20 Paragraph 2 Line 5, for the statement ‘In model 2, the variable satisfaction with the occupation (collected by SDO-OB) will be added’ to state clearly whether variables in model 1 are included in model 2 as well. Response: thanks for the comment, we have better clarified that sentence.
