# Supplementary material for: Study protocol for a non-randomised controlled trial: Community-based occupational therapy intervention on mental health for people with acquired brain injury (COT-MHABI)
Source: PLoS One. 2022 Oct 7;17(10):e0274193. doi: 10.1371/journal.pone.0274193 (PMC9543977; doi:10.1371/journal.pone.0274193)
Supplement: Attachment — Submitted filename: Response to reviewers.docx [file pone.0274193.s006.docx]

RESPONSE TO ACADEMIC EDITOR AND REVIEWER - PONE-D-21-30716R1

Note: author uses red for responses under the original comment

ACADEMIC EDITOR

1. We noticed that specifically the abstract needs some copy editing. PLOS ONE does not provide copy editing services, please ensure the use proper English language and grammar throughout the manuscript.

Response: Thank you very much for your comment, it has been very helpful, as we have been able to improve the quality of the abstract. Copy editing changes have been made and the translation has been revised.

2. We noticed that the sample size of your study has increased from 106 to 134. This does not agree with the registered trial protocol NCT04586842. Please comment on this and seek out advise if an amendment is required. If an amendment is required, please ensure it is granted before the manuscript is resubmitted. If no amendment is required please explain why.

Response: Thank you for the comment, as it was one of the changes made thanks to the reviewer's suggestion in the previous phase, we made a mistake in not updating the data in the ClinicalTrials registry. This is now fixed.

REVIEWER

1. Does the manuscript provide a valid rationale for the proposed study, with clearly identified and justified research questions?

The research question outlined is expected to address a valid academic problem or topic and contribute to the base of knowledge in the field.

Reviewer #1: Yes

2. Is the protocol technically sound and planned in a manner that will lead to a meaningful outcome and allow testing the stated hypotheses?

The manuscript should describe the methods in sufficient detail to prevent undisclosed flexibility in the experimental procedure or analysis pipeline, including sufficient outcome-neutral conditions (e.g. necessary controls, absence of floor or ceiling effects) to test the proposed hypotheses and a statistical power analysis where applicable. As there may be aspects of the methodology and analysis which can only be refined once the work is undertaken, authors should outline potential assumptions and explicitly describe what aspects of the proposed analyses, if any, are exploratory.

Reviewer #1: Partly

Response: Thank you for your comment. We are struggling to understand what specifically we could change or add in this section in order to improve the text. If you think a change is needed, can you tell us what you think is missing?

3. Is the methodology feasible and described in sufficient detail to allow the work to be replicable?

Descriptions of methods and materials in the protocol should be reported in sufficient detail for another researcher to reproduce all experiments and analyses. The protocol should describe the appropriate controls, sample size calculations, and replication needed to ensure that the data are robust and reproducible.

Reviewer #1: Yes

4. Have the authors described where all data underlying the findings will be made available when the study is complete?

The [PLOS Data policy](https://eur02.safelinks.protection.outlook.com/?url=https%3A%2F%2Fjournals.plos.org%2Fplosone%2Fs%2Fmaterials-and-software-sharing&data=05%7C01%7Cjosan.merchan%40uvic.cat%7Cc30cf119e48e4d571e1808da4b451c74%7C05bec0394f4f40879f2e1e71aa64c56e%7C0%7C0%7C637905057446275951%7CUnknown%7CTWFpbGZsb3d8eyJWIjoiMC4wLjAwMDAiLCJQIjoiV2luMzIiLCJBTiI6Ik1haWwiLCJXVCI6Mn0%3D%7C3000%7C%7C%7C&sdata=A%2BpGJwf3Ekj%2BjzgjOsv1WckOY5bHh3yLq5hoSOsmL%2F4%3D&reserved=0) requires authors to make all data underlying the findings described in their manuscript fully available without restriction, with rare exception, at the time of publication. The data should be provided as part of the manuscript or its supporting information, or deposited to a public repository. For example, in addition to summary statistics, the data points behind means, medians and variance measures should be available. If there are restrictions on publicly sharing data—e.g. participant privacy or use of data from a third party—those must be specified.

Reviewer #1: Yes

5. Is the manuscript presented in an intelligible fashion and written in standard English?

PLOS ONE does not copyedit accepted manuscripts, so the language in submitted articles must be clear, correct, and unambiguous. Any typographical or grammatical errors should be corrected at revision, so please note any specific errors here.

Reviewer #1: Yes

6. Review Comments to the Author

Please use the space provided to explain your answers to the questions above and, if applicable, provide comments about issues authors must address before this protocol can be accepted for publication. You may also include additional comments for the author, including concerns about research or publication ethics.

You may also provide optional suggestions and comments to authors that they might find helpful in planning their study.

(Please upload your review as an attachment if it exceeds 20,000 characters)

Reviewer #1: The authors have put in a great effort to address the comments.

Response: Thank you so much for your feedback and your dedication as a reviewer. We believe that this project is useful for the population profile for which it is designed, so we are enthusiastic about moving forward.

Minor comments

Line 358 - Typo error 'anaylisis'

Line 128-133 - Objectives to be placed at Line 127 (before Materials and Methods section)

Response: thank you so much for the comments. The suggested changes have been made.
